# The global burden of stroke attributable to high alcohol use from 1990 to 2021: An analysis for the global burden of disease study 2021

**DOI:** 10.1371/journal.pone.0328135

**Published:** 2025-07-14

**Authors:** Nannan Qian, Chengcheng Lu, Taohua Wei, Wenming Yang, Hui Han, Meixia Wang, Qiao Shi, Yulong Yang, Hu Xi, Wei He

**Affiliations:** 1 Department of Neurology, The First Affiliated Hospital of Anhui University of Chinese Medicine, Hefei, Anhui, China; 2 Anhui University of Chinese Medicine First Clinical Medical College, Hefei, Anhui, China; 3 Key Laboratory of Xin’An Medicine, Ministry of Education, Hefei, Anhui, China; 4 Graduate School, Anhui University of Chinese Medicine, Hefei, Anhui, China; 5 Clinical College of Anhui Medical University, Hefei, Anhui, China; 6 Center for Xin’an Medicine and Modernization of Traditional Chinese Medicine, Institute of Health and Medicine, Hefei Comprehensive National Science Center, Hefei, Anhui, China; Cedars-Sinai Heart Institute, UNITED STATES OF AMERICA

## Abstract

**Background:**

Stroke, a leading global cause of death and disability, has high alcohol consumption as a significant modifiable risk factor. Despite the known association, the global spatiotemporal burden and changing relationship between high alcohol use and stroke subtypes remain inadequately characterized. This study quantifies the global, regional, and national burden of stroke attributable to high alcohol intake from 1990 to 2021.

**Methods:**

Utilizing data from the Global Burden of Disease (GBD) 2021 study, we analyzed deaths, disability-adjusted life years (DALYs), years lived with disability (YLDs), and years of life lost (YLLs) for stroke attributable to high alcohol use. Metrics were age-standardized rates and stratified by sex, age, sociodemographic index (SDI), GBD region, and stroke subtype (ischemic stroke, intracerebral hemorrhage). Estimated annual percentage change (EAPC) quantified trends. Frontier analysis, decomposition analysis, and cross-country inequality analysis assessed socioeconomic disparities.

**Results:**

Globally, ASMR decreased by 40.28% (from 7.20 [95% UI 1.40–14.66] to 4.30 [1.00–8.39] per 100,000, EAPC = −1.81) and ASDR declined from 154.83 [33.98–299.48] to 97.89 [23.83–187.71] per 100,000 (EAPC = −1.63). While age-standardized YLL rates markedly improved (EAPC = −1.75), age-standardized YLD rates declined minimally (EAPC = −0.25), indicating persistent long-term disability burden. Significant disparities existed: males consistently bore a higher burden than females, though female ASMR declined more significantly (55.86% vs. 34.25%). High SDI regions showed substantial declines (e.g., ASMR EAPC = −3.28), but low-middle SDI regions experienced increasing ASMR (EAPC = 0.37) and ASDR (EAPC = 0.43), driven by rising YLDs and YLLs. Southeast Asia had the largest ASMR increase (EAPC = 2.86). National burdens were highest in Bulgaria, North Macedonia, and Vietnam. Ischemic stroke burden showed reducing socioeconomic inequality, but intracerebral hemorrhage burden increasingly concentrated in disadvantaged populations (SII = −47.40, CII = −0.19 in 2021). Frontier analysis identified Vietnam, Bulgaria, and Laos with the largest unrealized health potential. Decomposition revealed global DALYs increases were driven by population aging (92.5%) and growth (149.3%), partially offset by reduced age-specific rates (−141.8%).

**Conclusion:**

Global stroke mortality attributable to high alcohol use declined significantly from 1990 to 2021, reflecting progress in prevention and acute care. However, minimal improvement in disability burden reveals critical gaps in long-term management and rehabilitation, creating a “survival-disability paradox.” Profound disparities persist across genders, regions, SDI levels, and stroke subtypes. Targeted policies addressing excessive alcohol consumption, tailored to regional contexts and focused on both prevention and comprehensive post-stroke care, are urgently needed, particularly in low-middle SDI regions and Southeast Asia, to mitigate disability and health inequities.

## Introduction

Stroke, the second leading cause of death globally, is a clinical syndrome characterized by neurological deficits resulting from acute cerebrovascular lesions. It primarily comprises ischemic stroke (85%) and Intracerebral hemorrhage (15%) [1,2]. Ischemic stroke occurs due to cerebrovascular obstruction leading to ischemic necrosis of brain tissue, while Intracerebral hemorrhage results from vascular rupture causing brain parenchyma or subarachnoid hemorrhage. Collectively, these conditions account for approximately 6.2 million deaths annually, with a disability rate of up to 50%, and incur over US$720 billion in direct medical costs and productivity losses [2,3]. Despite advancements in diagnostic and treatment methods such as intravenous thrombolysis, intravascular thrombectomy, and multimodal rehabilitation significantly reducing acute mortality, secondary prevention remains constrained by inadequate control of risk factors (exemplified by the global hypertension control rate of <40%), particularly the absence of precise targets for preventing alcohol-related stroke [2,4]. Research indicates that excessive alcohol consumption elevates stroke risk by inducing hypertension, coagulation abnormalities, and endothelial dysfunction. However, the spatiotemporal heterogeneity of its global disease burden and its associative mechanisms with stroke subtypes have not been systematically elucidated.

Recent research has elucidated a J-shaped relationship between alcohol consumption and stroke risk. Daily ethanol intake exceeding 40 grams can increase the risk of ischemic stroke by 35% and Intracerebral hemorrhage by 82% [5,6]. However, current studies exhibit notable limitations. First, the research exhibits a regional bias, as the majority of evidence originates from European and American populations, thereby overlooking the distinct characteristics of Asia and Africa, where alcohol consumption is substantial. For instance, the potential effects of methanol impurities in Southeast Asian rice wine on the vascular endothelium remain underexplored, and drinking patterns in Vietnam reveal that alcohol consumption is significantly more prevalent among men than women across all age groups [7]. Secondly, there is insufficient mechanistic analysis, particularly regarding the association between alcohol metabolism gene ALDH2 mutation and the high incidence of Intracerebral hemorrhage in East Asia [4]. These research gaps hinder the development of region-specific prevention and control strategies, such as implementing graduated alcohol tax pricing or establishing alcohol restriction guidelines for individuals with ALDH2 mutations in Eastern Europe [6,8].

Drawing from the 2021 GBD database, this study employed the standardized attributable fraction method to quantify ASMR, ASDR, age-standardized rate of YLDs and age-standardized rate of YLLs of stroke attributed to high alcohol use (>60 g/day for males and >40 g/day for females) from 1990 to 2021 [9,10]. The GBD hierarchical Bayesian model (DISMOD-MR 2.1) was utilized to address data heterogeneity, systematically elucidate burden disparities across different SDI regions, and innovatively analyze the interactive effects of alcohol with stroke subtypes, sex, and age [11]. The study objectives are to: First, delineate the global spatial distribution of alcohol-related stroke; Secondly, To explore the potential impact of biological mechanisms (e.g., ALDH2 gene polymorphisms) and social factors (including regional drinking culture) on age- and sex-specific burdens; moreover, By analyzing regional variations in stroke incidence and outcomes, this study investigates the impact of alcohol control policies on the burden of stroke, thereby offering potential optimized policy recommendations for the WHO Global Framework for Action on Alcohol Control (2022–2030).

## Methods

### Study design and data sources

The 2021 GBD analysis draws from 100,983 data sources, providing a comprehensive dataset encompassing incidence, prevalence, YLDs, YLLs, and DALYs for 371 diseases and injuries across 204 countries and territories and 811 subnational areas from 1990 to 2021 [11]. This study examines repeated cross-sectional data on deaths, DALYs, YLDs, YLLs, and their corresponding age-standardized rates (ASRs) with 95% uncertainty intervals (UIs). In a given population, incidence denotes the total number of new cases within a specific time frame, while mortality represents the proportion of deaths due to a particular disease relative to the entire population during that period. YLDs were calculated by multiplying the cause-age-sex-location-year prevalence of each disease and injury by its associated disability weight. YLLs were derived by multiplying the standard life expectancy at death by the cause-age-sex-location-year number of deaths. DALYs comprise the sum of YLDs and YLLs. The 95% uncertainty intervals for all final estimates were determined by generating 2.5th and 97.5th percentile values from 500 samples.

### Socioeconomic index

The 2021 GBD database utilizes the SDI to evaluate and compare socioeconomic development levels across countries and regions. This composite index integrates three key factors: income level, education level, and fertility rate, weighted accordingly. The resulting SDI categorizes countries or regions into five distinct levels: low, low-middle, middle, high-middle, and high. This classification methodology facilitates the understanding of variations in health outcomes and disease burden among countries or regions at different developmental stages. In this study, ASMR, ASDR, age-standardized rate of YLDs, and age-standardized rate of YLLs were analyzed across various SDI levels. Subsequently, the EAPC of these indicators was examined.

### Statistical analysis

The Institute for Health Metrics and Evaluation (IHME) developed DISMOD-MR 2.1, a Bayesian hierarchical modeling framework designed to produces internally consistent estimates of prevalence, incidence, and mortality, stratified by sex, region, year, and age group [12]. In areas with limited original epidemiological data, DisMod-MR 2.1 leverages data from higher hierarchical levels as prior information to estimate parameters for lower levels. For specific causes, the Space-Time Gaussian Process Regression (ST-GPR) model serves as an alternative estimation method. For nonfatal causes, prevalence and incidence are further disaggregated into sequela-specific estimates based on severity levels. Sequela categories for nonfatal causes vary from asymptomatic to severe, depending on the disease type. For most nonfatal causes, the distribution of cases across sequela categories is determined using Medical Expenditure Panel Survey (MEPS) analysis. Crude YLD rates are calculated by multiplying sequela-specific prevalence estimates by their corresponding disability weights. The quantification of mortality, DALYs, YLDs, and YLLs, along with their respective ratios, provides insight into the burden of stroke. Each rate is reported per 100,000 individuals and includes a 95% uncertainty interval based on the GBD methodology. Furthermore, the EAPC was employed for dynamic analysis of stroke to identify temporal trends in disease burden. the EAPC and its 95%UI are calculated as follows: First, the natural logarithm of the age-standardized rate (ASR) is computed for each time point in the series. A linear regression model is then constructed to relate the transformed values (ln(ASR)) to the time variable (t), yielding a slope coefficient (β₁). The EAPC is derived by exponentiating β₁, multiplying by 100, and interpreting the result as a percentage change (Formula:$EAPC=100\times (e^{\beta_{1}}-1)$), where positive/negative values indicate increasing/decreasing trends. The 95%UI is calculated using the standard error of β₁ (SEβ₁) (Formula: $95\;\%UI=100\times (e^{\beta_{1}\pm 1.96\times SE_{\beta_{1}}}-1)$). Trends are considered statistically significant (p < 0.05) if the 95%UI excludes zero. This approach quantifies longitudinal trends in health metrics while accounting for uncertainty [13]. To achieve a more comprehensive understanding of the underlying factors influencing variations in high alcohol use-related stroke DALYs from 1990 to 2021, we performed a decomposition analysis that accounted for population growth, aging, and epidemiological transitions. Furthermore, a frontier analysis was conducted to explore the correlation between high alcohol use-related stroke DALYs and the SDI. A nonlinear frontier was constructed to represent the minimum achievable DALYs at a given level of development. The effective difference was defined as the gap between the observed ASDR for strokes and the ASDR-DALYs relative to this frontier, highlighting the unrealized health improvements considering the current developmental status of each region or country. This study employed the slope index of inequality (SII) and the concentration index, as defined by the World Health Organization (WHO), to evaluate absolute and relative inequalities in disease burden. These indices served as quantitative measures to analyze disparities in stroke distribution across countries and territories, providing a thorough assessment of health inequalities [14]. These figures were generated using the R software package (version 4.4.2) and JD_GBDR (V2.2, provided by Jingding Medical Technology Co., Ltd.).

## Results

### Global burden of stroke due to high alcohol use

**Fig 1:**
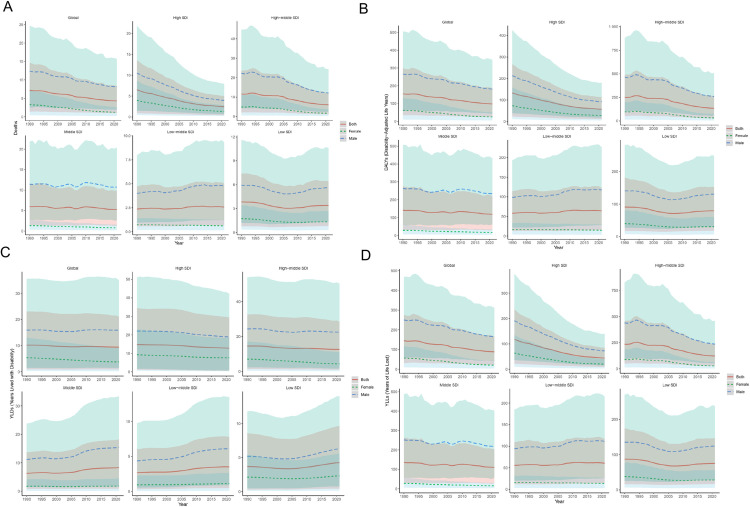
ASMR (A), ASDR (B), Age-Standardized Rate of YLDs (C), and Age-Standardized Rate of YLLs (D) of high alcohol use-related Stroke in both sexes combined in global and five SDI regions, 1990–2021. ASMR, age-standardized mortality rate; DALYs, disability-adjusted life years; ASDR, age-standardized rate of DALYs; YLDs, years lived with disability; YLLs, years of life lost; SDI, socio-demographic index.

Between 1990 and 2021, the global age-standardized mortality rate (ASMR), age-standardized DALYs rate (ASDR), age-standardized rate of YLDs, and age-standardized rate of YLLs for stroke attributable to high alcohol use exhibited a general decline. The ASMR and age-standardized rate of YLLs demonstrated the most substantial decrease, while the age-standardized rate of YLDs showed a more gradual reduction (Fig 1; S1 Fig). The global stroke ASMR attributable to high alcohol use decreased by 40.28%, from 7.20 per 100,000 people (95% UI, 1.40 to 14.66) in 1990 to 4.30 per 100,000 people (95% UI, 1.00 to 8.39) in 2021. The EAPC was −1.81 (95% CI, −1.88 to −1.75). Throughout this period, the ASMR for males consistently exceeded that of females. However, the mortality rate for females demonstrated a more pronounced decrease of 55.86% compared to 34.25% in males (Fig 1). The ASDR decreased from 154.83 per 100,000 in 1990 (95% UI, 33.98 to 299.48) to 97.89 per 100,000 in 2021 (95% UI, 23.83 to 187.71), with an EAPC of −1.63 (95% CI, −1.70 to −1.56). age-standardized rate of YLLs declined from 144.63 per 100,000 people (95% UI, 31.98 to 275.44) in 1990 to 88.44 per 100,000 people (95% UI, 22.35 to 167.25) in 2021, demonstrating an EAPC of −1.75 (95% CI, −1.82 to −1.67; Fig 1; S1 Table). Conversely, age-standardized rate of YLDs exhibited a gradual decline, decreasing from 10.20 per 100,000 individuals (95% UI, 0.89 to 23.02) in 1990 to 9.45 per 100,000 individuals (95% UI, 0.85 to 21.18) in 2021, with an EAPC of −0.25 (95% CI, −0.29 to −0.20; Fig 1; S1 Table).

### Regional burden of stroke due to high alcohol use

From 1990 to 2021, global high alcohol use-related stroke burden exhibited significant disparities when stratified by SDI. High SDI regions demonstrated the most substantial improvement. The ASMR decreased substantially from 6.50 per 100,000 (95% UI: 0.91–13.60) to 2.49 per 100,000 (95% UI: 0.45–5.05), representing a 61.7% reduction with an EAPC of −3.28 (95% CI: −3.40 to −3.17). Concurrently, the ASDR declined from 132.55 per 100,000 (95% UI: 22.99–266.22) to 57.25 per 100,000 (95% UI: 10.71–115.15) (EAPC = −2.88). This significant reduction, especially in age-standardized rate of YLLs (EAPC = −3.34), reflects the effectiveness of comprehensive interventions such as widespread early screening and statin utilization. However, the smaller decline in age-standardized rate of YLDs (EAPC = −0.52) indicates an ongoing need for improved chronic disease management (S1 Table).

High-middle SDI regions achieved moderate progress, with ASMR decreasing from 11.56 per 100,000 (95% UI: 2.06–23.67) to 6.03 per 100,000 (95% UI: 1.19–12.28) (EAPC = −2.45) and ASDR falling from 250.70 per 100,000 (95% UI: 51.62–489.75) to 134.48 per 100,000 (95% UI: 30.10–266.02) (EAPC = −2.39). Notably, the age-standardized rate of YLLs decline (EAPC = −2.53) outpaced the minimal reduction in age-standardized rate of YLDs (EAPC = −0.49), suggesting better acute care efficacy compared to post-stroke rehabilitation and long-term functional management, potentially due to resource limitations (S1 Table).

Middle SDI regions displayed a pattern of persistent burden. ASMR declined only marginally from 5.97 per 100,000 (95% UI: 1.39–11.48) to 5.24 per 100,000 (95% UI: 1.28–9.89) (12.2% reduction; EAPC = −0.33), while ASDR decreased modestly from 141.18 per 100,000 (95% UI: 31.07–275.13) to 118.52 per 100,000 (95% UI: 30.96–219.97) (EAPC = −0.45). A concerning increase in age-standardized rate of YLDs (EAPC = 1.06) contrasted with a small age-standardized rate of YLLs decline (EAPC = −0.53), possibly attributable to aging populations and insufficient chronic disease management (S1 Table).

Low-middle SDI regions were the only tier experiencing an increase in both mortality and disability burden. ASMR rose from 2.36 per 100,000 (95% UI: 0.57–4.74) to 2.56 per 100,000 (95% UI: 0.63–5.02) (EAPC = 0.37), and ASDR increased from 58.03 per 100,000 (95% UI: 12.79–114.64) to 64.04 per 100,000 (95% UI: 14.92–123.09) (EAPC = 0.43). This upward trend was driven by concurrent increases in both age-standardized rate of YLDs (EAPC = 1.06) and age-standardized rate of YLLs (EAPC = 0.40), likely linked to rising diabetes prevalence, low antiplatelet medication use, and weak emergency response systems (S1 Table).

Low SDI regions saw minor reductions in ASMR [from 3.84 per 100,000 (95% UI: 0.79–7.42) to 3.38 per 100,000 (95% UI: 0.82–6.46); EAPC = −0.51] and ASDR [from 91.01 per 100,000 (95% UI: 17.08–181.35) to 79.17 per 100,000 (95% UI: 18.21–153.35); EAPC = −0.58]. A paradoxical increase in age-standardized rate of YLDs (EAPC = 0.50) alongside declining age-standardized rate of YLLs (EAPC = −0.63) suggests improved survival but rising disability prevalence, possibly indicating inadequate rehabilitation services (S1 Table).

Significant geographical disparities in disease burden trajectories were observed across GBD regions. In Australasia, substantial progress was noted, with the ASMR decreasing by 61.3% from 5.61 to 2.17 per 100,000 (EAPC = −3.12), and the ASDR declining from 101.00 to 41.63 per 100,000 (EAPC = −2.93). Western Europe demonstrated even more pronounced improvements, achieving a 70.9% reduction in ASMR (EAPC = −4.12) and a 67.7% decrease in ASDR (EAPC = −3.80). Tropical Latin America also showed consistent advancements, with ASMR falling from 6.24 to 2.48 per 100,000 (EAPC = −3.02) (S1 Table).

In contrast, East Asia and Central Asia exhibited mixed patterns. East Asia experienced a modest decline in ASMR from 10.59 to 7.79 per 100,000 (EAPC = −0.92), but ASDR increased (EAPC = 1.03). Central Asia saw a reduction in ASDR from 160.00 to 120.51 per 100,000 (EAPC = −1.34), yet maintained a persistently high age-standardized rate of YLDs at 10.36 per 100,000, reflecting gaps in chronic disease management (S1 Table).

Alarming trends persisted in three regions. Southeast Asia experienced paradoxical increases in both ASMR (from 2.40 to 5.08 per 100,000; EAPC = 2.86) and ASDR (105.1% rise; EAPC = 2.73), likely associated with a doubling of diabetes prevalence (from 8% to 16%) and limited emergency care coverage (<30%). South Asia displayed a “high morbidity-low mortality” paradox, with ASDR rising from 38.01 to 46.10 per 100,000 (EAPC = 0.83) despite a 7.3% reduction in ASMR (EAPC = 0.92), potentially linked to increased antiplatelet therapy utilization (from 15% to 45%). In Western Sub-Saharan Africa, minimal improvement in ASMR was observed (from 7.29 to 6.48 per 100,000; EAPC = −0.53). However, diagnostic limitations, including CT/MRI accessibility below 10% and estimated misdiagnosis rates exceeding 50%, suggest that the true disease burden may be substantially underestimated (S1 Table).

### National burden of stroke due to high alcohol use

**Fig 2:**
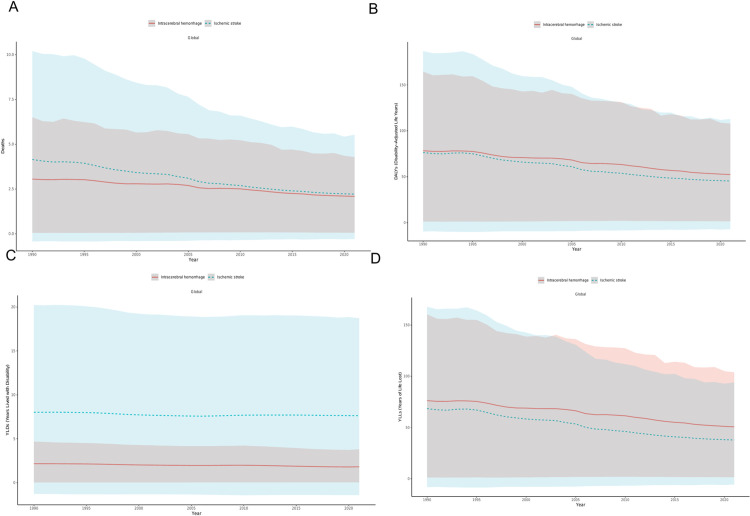
ASMR (A), ASDR (B), Age-Standardized Rate of YLDs (C), and Age-Standardized Rate of YLLs (D) of high alcohol use-related Stroke in both sexes combined in global in 2021. ASMR, age-standardized mortality rate; DALYs, disability-adjusted life years; ASDR, age-standardized rate of DALYs; YLDs, years lived with disability; YLLs, years of life lost.

In 2021, the five countries with the highest ASMR for stroke due to high alcohol use were Bulgaria [18.16 (95% UI, 2.30 to 38.39) per 100,000], North Macedonia [17.50 (95% UI, 1.74 to 38.61) per 100,000], Viet Nam [16.22 (95% UI, 4.02 to 29.11) per 100,000], Montenegro [15.19 (95% UI, 2.94 to 30.10) per 100,000], and Namibia [12.28 (95% UI, 2.68 to 24.77) per 100,000]. Conversely, Morocco [0.08 (95% UI, 0.01–0.19) per 100,000], Kuwait [0.02 (95% UI, 0.00–0.07) per 100,000], Sudan [0.00 (95% UI, −0.00–0.00) per 100,000], Somalia [0.00 (95% UI, 0.00–0.00) per 100,000], and Mauritania [0.00 (95% UI, 0.00–0.00) per 100,000] exhibited the lowest ASMR (Fig 2; S2 Table).

The five countries with the highest ASDR are Vietnam [378.50 (95% UI, 84.09, 688.25) per 100,000], Bulgaria [369.04 (95% UI, 58.16, 731.32) per 100,000], Lao People’s Democratic Republic [310.31 (95% UI, 77.64, 589.73) per 100,000], North Macedonia [301.81 (95% UI, 43.13, 623.58) per 100,000], and Nauru [285.97 (95% UI, 49.32, 608.13) per 100,000]. Conversely, the five countries with the lowest ASDR were Afghanistan [2.47 (95% UI, 0.25, 6.36) per 100,000], Kuwait [0.56 (95% UI, −0.01, 1.86) per 100,000], Somalia [0.00 (95% UI, 0.00, 0.00) per 100,000], Mauritania [0.00 (95% UI, 0.00, 0.00) per 100,000], and Sudan [−0.01 (95% UI, −0.11, 0.03) per 100,000] (Fig 2; S2 Table). The age-standardized rate of YLDs were highest in Bulgaria [23.68 (95% UI, 0.42, 53.44) per 100,000] and lowest in Sudan [−0.00 (95% UI, −0.02, 0.00) per 100,000] (Fig 2; S2 Table). The age-standardized rate of YLLs were highest in Vietnam [360.35 (95% UI, 82.87, 660.63) per 100,000] and lowest in Sudan [−0.01 (95% UI, −0.09, 0.03) per 100,000] (Fig 2; S2 Table).

**Fig 3:**
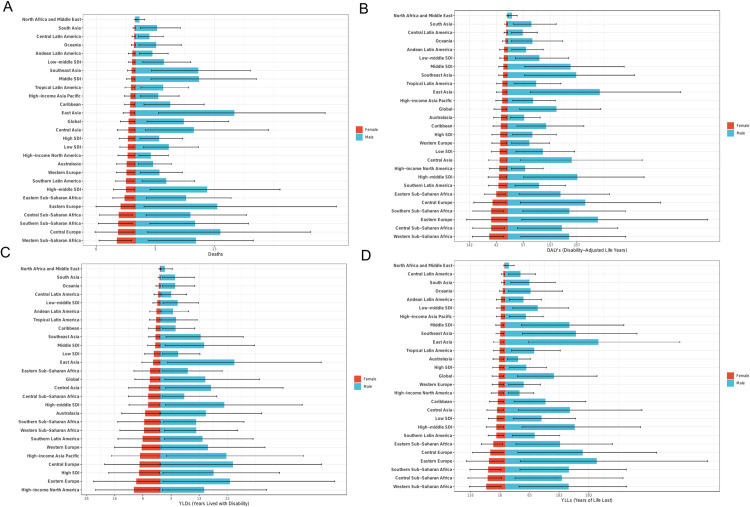
ASMR (A), ASDR (B), Age-Standardized Rate of YLDs (C), and Age-Standardized Rate of YLLs (D) of high alcohol use-related Stroke in males and females across 21 GBD regions and SDI regions in 2021. ASMR, age-standardized mortality rate; DALYs, disability-adjusted life years; ASDR, age-standardized rate of DALYs; YLDs, years lived with disability; YLLs, years of life lost.

The 2021 data revealed substantial regional variations in ASMR, ASDR, age-standardized rate of YLDs, and age-standardized rate of YLLs for stroke attributable to high alcohol use across the 21 GBD regions and the five SDI regions (Fig 3; S1 Table; S1 Fig).

East Asia and Central Europe exhibited the highest ASMR, while Central Latin America and North Africa and the Middle East demonstrated the lowest. Between 1990 and 2021, ASMR increased in three of the 21 GBD regions and in three of the five SDI regions. The most substantial increases were observed in Southeast Asia [EAPC = 2.86 (95% CI, 2.54 to 3.17)] and Low-middle SDI [EAPC = 0.37 (95%CI, 0.28 to 0.46)]. Conversely, the most significant mortality declines were recorded in High-income Asia Pacific [EAPC = −4.63 (95%CI, −4.81 to −4.45)] and High SDI [EAPC = −3.28 (95%CI, −3.40 to −3.17)] (Fig 3; S1 Table; S1 Fig).

Regarding ASDR, 19 GBD regions and 4 SDI regions experienced declines. The most substantial decreases were observed in High-income Asia Pacific [EAPC = −2.88 (95% CI, −2.99 to −2.77)] and High SDI [EAPC = −4.03 (95% CI, −4.21 to −3.85)]. Conversely, the most significant increases were noted in Southeast Asia [EAPC = 2.73 (95% CI, 2.44 to 3.02)] and Low-middle SDI [EAPC = 0.43 (95% CI, 0.34 to 0.52)] (S1 Table; S1 Fig).

Age-standardized rate of YLDs in the seven GBD regions and three SDI regions exhibited an upward trend, with the most substantial increases observed in Southeast Asia [EAPC = 2.60 (95%CI, 2.42 to 2.78)] and Low-middle SDI [EAPC = 1.06 (95%CI, 0.97 to 1.16)]. Conversely, 14 GBD regions and 2 SDI regions demonstrated a downward trend, with the most significant decreases noted in Southern Latin America [EAPC = −2.00 (95%CI, −2.13 to −1.87)] and High SDI [EAPC = −0.52 (95%CI, −0.56 to −0.47)]. In 2021, the highest age-standardized rate of YLDs were recorded in Central Europe [15.35 (95% UI, −0.02 to 35.68) per 100,000] and High SDI [12.83 (95% UI, 0.65 to 29.37) per 100,000], while the lowest were observed in North Africa and the Middle East [0.89 (95% UI, 0.06 to 2.44) per 100,000] and Low-middle SDI [3.51 (95% UI, 0.49 to 7.79) per 100,000] (S1 Table; S1 Fig).

Age-standardized rate of YLLs exhibited an upward trend in two GBD regions and one SDI region, with the most pronounced increases observed in Southeast Asia [EAPC = 2.74 (95%CI, 2.44 to 3.04)] and Low-middle SDI [EAPC = 0.40 (95%CI, 0.31 to 0.49)]. Conversely, the most significant decreases were noted in High-income Asia Pacific [EAPC = −4.61 (95%CI, −4.79 to −4.43)] and High SDI [EAPC = −3.34 (95%CI, −3.46 to −3.22)]. In 2021, the highest age-standardized rate of YLLs were recorded in East Asia [156.61 (95% UI, 40.16 to 295.44) per 100,000] and High-middle SDI [121.78 (95% UI, 29.66 to 239.58) per 100,000], while the lowest were observed in High SDI [44.42 (95% UI, 9.23 to 86.29) per 100,000] and Central Latin America [26.99 (95% UI, 6.47 to 53.91) per 100,000] (S1 Table; S1 Fig).

### Trends in gender and age distribution of stroke due to high alcohol use

In 2021, the ASMR, ASDR, age-standardized rate of YLDs, and age-standardized rate of YLLs were consistently higher for males compared to females across the world, 21 GBD regions, and 5 SDI regions (Fig 3; S3 Table).

Western Sub-Saharan Africa exhibited the highest ASMR, ASDR, and age-standardized rate of YLLs for females among the 21 GBD regions and 5 SDI regions. However, High-income North America recorded the highest age-standardized rate of YLDs for females. Conversely, North Africa and the Middle East reported the lowest ASMR, ASDR, age-standardized rate of YLDs, and age-standardized rate of YLLs for females (Fig 3; S3 Table).

**Fig 4:**
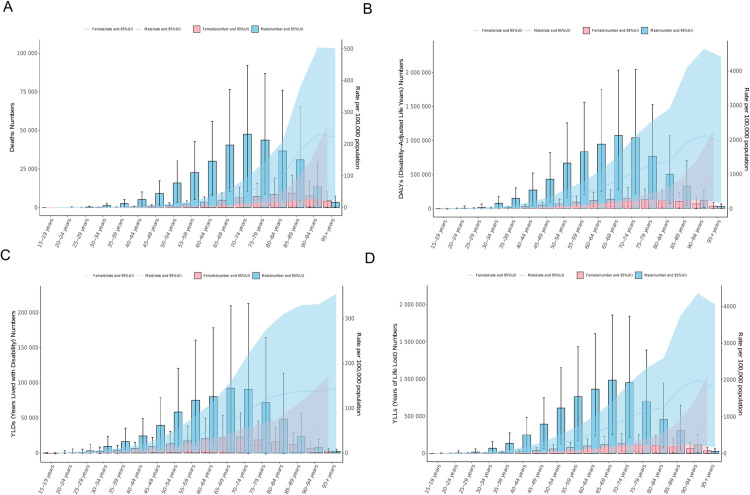
The age-specific burden of high alcohol use-related Stroke, death and ASMR (A), DALYs and ASDR (B), YLDs and Age-Standardized Rate of YLDs (C), YLLs and Age-Standardized Rate of YLLs (D) globally in 2021. ASMR, age-standardized mortality rate; DALYs, disability-adjusted life years; ASDR, age-standardized rate of DALYs; YLDs, years lived with disability; YLLs, years of life lost; UI, uncertainty interval.

In 2021, the age-related patterns of stroke mortality and morbidity due to high alcohol use exhibited a characteristic rise-and-fall trajectory. For males, the peak in stroke deaths attributable to high alcohol use occurred in the 70–74 year age group, while for females, this peak was observed in the 85–89 year age group. The DALYs reached their maximum for males aged 65–69 years and for females aged 70–74 years. Similarly, both YLDs and YLLs peaked for males in the 65–69 year age group and for females in the 70–74 year age group. These trends are visually represented in Fig 4 and further detailed in S4 Table.

In 2021, the ASMR, ASDR, and age-standardized rate of YLLs for males exhibited a rapid increase initially, followed by a decline after 94 years of age. Conversely, the age-standardized rate of YLDs continued to increase. For females, a consistent increase was observed in ASMR, ASDR, age-standardized rate of YLDs, and age-standardized rate of YLLs (Fig 4; S5 Table).

### Trends in stroke distribution due to high alcohol use

**Fig 5:**
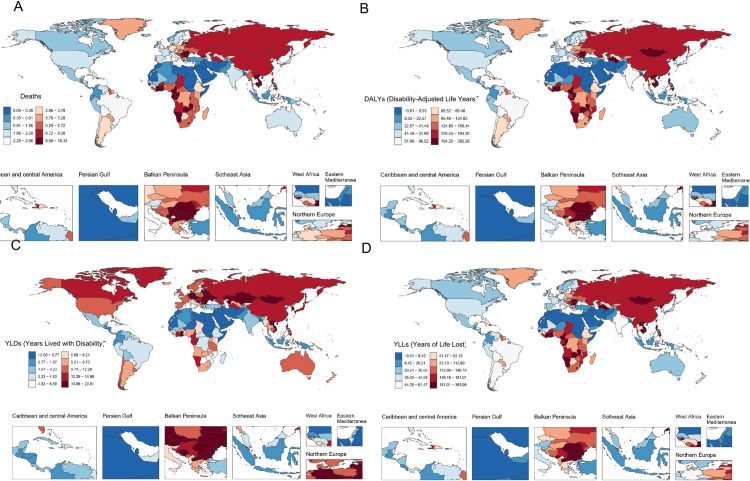
ASMR (A), ASDR (B), Age-Standardized Rate of YLDs (C), and Age-Standardized Rate of YLLs (D) for two types of high alcohol use-related Stroke in both sexes combined globally, 1990–2021. ASMR, age-standardized mortality rate; DALYs, disability-adjusted life years; ASDR, age-standardized rate of DALYs; YLDs, years lived with disability; YLLs, years of life lost.

The GBD database categorizes stroke into two types: ischemic stroke and intracerebral hemorrhage. Between 1990 and 2021, the ASMR, ASDR, and age-standardized rate of YLLs for both ischemic stroke and intracerebral hemorrhage demonstrated a declining trend. However, the age-standardized rate of YLDs remained relatively stable during this period (Fig 5; S6–S9 Tables).

Among the 21 GBD regions, High-income Asia Pacific, High-income North America, Central Europe, Eastern Europe, Western Sub-Saharan Africa, and Eastern Sub-Saharan Africa ranked highest in ASMR, ASDR, age-standardized rate of YLDs and age-standardized rate of YLLs of ischemic stroke. High-income Asia Pacific, Central Europe, Eastern Europe and East Asia ranked highest in ASMR, ASDR, age-standardized rate of YLDs and age-standardized rate of YLLs of intracerebral hemorrhage (S2 Fig).

### Cross-country inequality analysis, Frontier analysis and decomposition analysis

**Fig 6:**
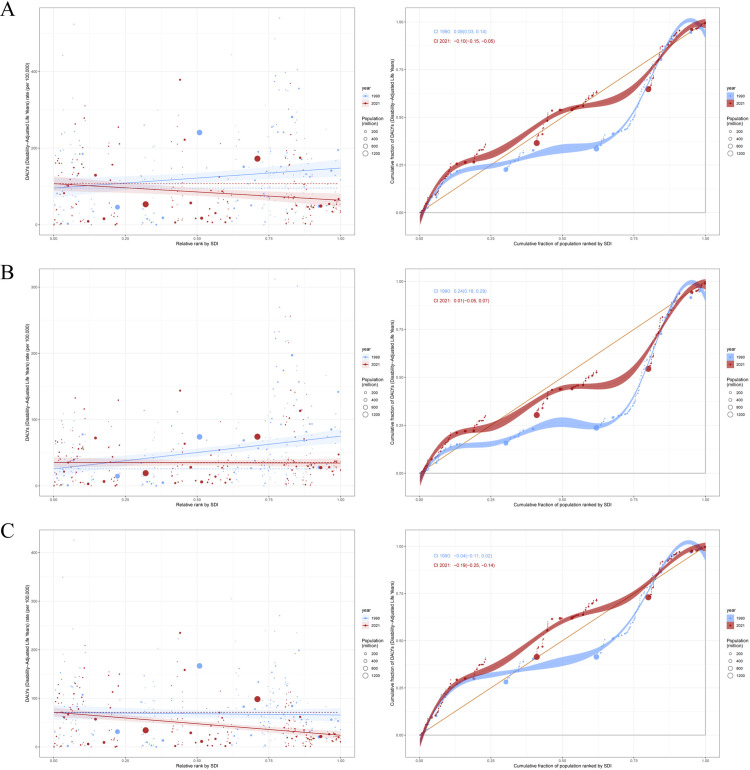
Health inequality regression curves and concentration curves for the DALYs of two types of high alcohol use-related Stroke worldwide, 1990 and 2021. **(A)** high alcohol use-related Stroke. **(B)** high alcohol use-related Ischemic stroke. **(C)** high alcohol use-related Intracerebral hemorrhage.

**Fig 7:**
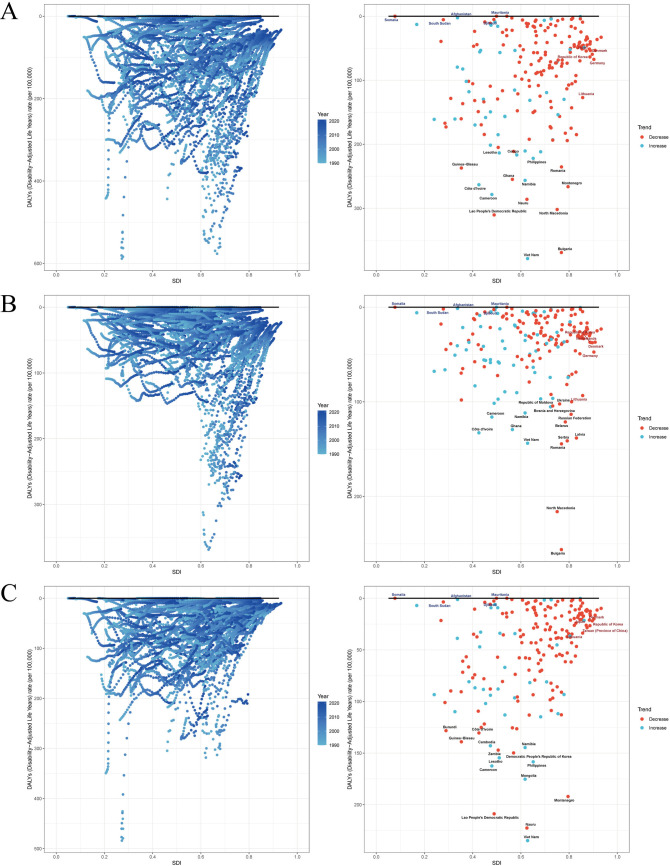
Frontier analysis exploring the relationship between SDI and DALYs for two types of high alcohol use-related Stroke in 204 countries and territories. **(A)** high alcohol use-related Stroke. **(B)** high alcohol use-related Ischemic stroke. **(C)** high alcohol use-related Intracerebral hemorrhage.

**Fig 8:**
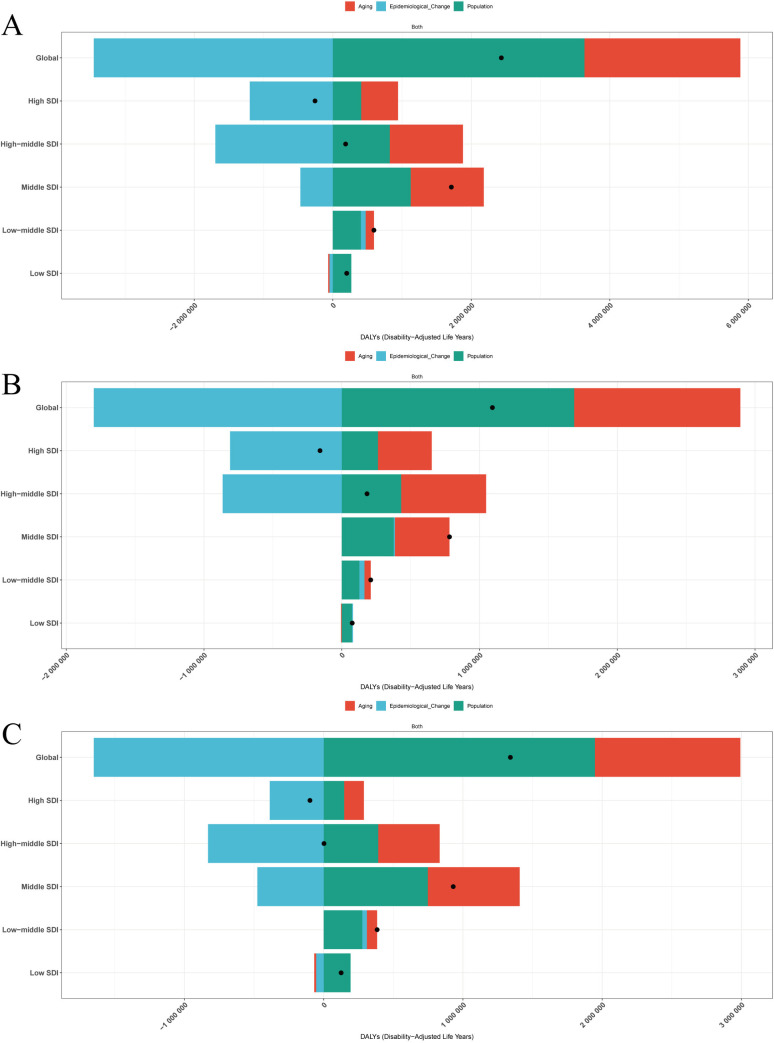
Population-level determinant changes in aging, population growth, and epidemiological changes for two types of high alcohol use-related Stroke DALYs globally and in various SDI regions from 1990 to 2021. **(A)** high alcohol use-related Stroke. **(B)** high alcohol use-related Ischemic stroke. **(C)** high alcohol use-related Intracerebral hemorrhage.

From 1990 to 2021, the global burden of stroke attributable to high alcohol use underwent marked socioeconomic and epidemiological transitions, with distinct patterns observed across stroke subtypes. For overall stroke, the SII shifted from 52.31 in 1990 (95% CI: 13.06–91.55) to −43.36 in 2021 (95% CI: −72.69–-14.03), signifying a reversal in DALYs burden from higher to lower socioeconomic status groups. This pattern was corroborated by the CII, which decreased from 0.08 (95% CI: 0.03–0.14) to −0.10 (95% CI: −0.15–-0.05), demonstrating a redistribution toward socioeconomically disadvantaged populations (Fig 6). Frontier analysis identified Vietnam with an efficiency difference of 378.52, Bulgaria at 369.06, and Laos at 310.31 as having the most substantial avoidable DALYs gaps relative to their socioeconomic development index levels, compared to Somalia and Mali approaching theoretical optimum values near zero (Fig 7). Global DALYs trend decomposition from 1990 to 2021 revealed a net increase of 243,000 DALYs per 100,000 population, primarily driven by population aging at 92.5% and population growth at 149.3%, despite a 141.8% reduction in age-specific incidence rates through epidemiological improvements (Fig 8).

For ischemic stroke, the socioeconomic gradient diminished over time. The SII decreased from 49.47 (95% CI: 31.03–67.90) in 1990 to an insignificant −0.97 (95% CI: −12.80–10.87) by 2021. Concurrently, the CII declined from 0.24 (95% CI: 0.18–0.29) to 0.01 (95% CI: −0.05–0.07), indicating improved equity in distribution (Fig 6). Regional Frontier analysis exposed Eastern European anomalies, with Bulgaria showing excess DALYs of 256.14, North Macedonia 216.03, and Romania 144.38 relative to their socioeconomic development index, contrasting with high-development nations like Denmark achieving near-optimal performance at 21.88 (Fig 7). Decomposition analysis showed high-development regions attained a 257,000 DALYs reduction through 466.6% epidemiological benefits, whereas middle-development countries experienced a 1.71 million DALYs increase driven by 61.7% population aging and 65.7% economic growth (Fig 8).

Intracerebral hemorrhage exhibited widening socioeconomic disparities. The SII intensified from −5.18 (95% CI: −27.52–17.17) to −47.40 (95% CI: −64.27–-30.54) between 1990 and 2021, accompanied by a ninefold increase in absolute burden for lower socioeconomic groups. The CII for hemorrhage deteriorated from −0.04 (95% CI: −0.11–0.02) to −0.19 (95% CI: −0.25–-0.14), confirming disproportionate burden concentration. Vietnam and Nauru demonstrated the most pronounced efficiency gaps exceeding 200, contrasting with Somalia and South Sudan nearing theoretical minima below 3.58 (Fig 6). Regional decomposition revealed high-development countries achieved a 99,020 DALYs reduction through 391.3% epidemiological advances, while middle-development regions saw a 383,506 DALYs surge primarily from 72.3% population growth. Low-development areas exhibited a paradoxical 124,852 DALYs decrease attributed to negative impacts from aging at −11.3% and epidemiological transitions at −43.1% (Figs 7 and 8).

These findings underscore the divergent epidemiological trajectories: ischemic stroke burden stabilizes across socioeconomic strata, while intracerebral hemorrhage disproportionately affects disadvantaged populations. The systemic inefficiency in middle-development nations and persistent treatment disparities necessitate targeted interventions addressing modifiable risk factors and healthcare access barriers.

## Discussion

This study unveils the intricate evolutionary characteristics of the stroke disease burden associated with high alcohol use. The notable decrease in ASMR and age-standardized rate of YLLs globally (1990−2021) may be attributed to the widespread adoption of alcohol control policies, advancements in thrombolytic and thrombectomy technologies, and improvements in secondary prevention systems, aligning with the overall trend of declining stroke mortality worldwide [15–17]. However, the slower improvement in ASDR and age-standardized rate of YLDs warrants attention, indicating that current intervention measures have limited efficacy in reversing disability status. It is particularly significant that the increased stroke treatment rate and subsequent expansion of the survivor group may objectively increase the number of years lived with disability. This “survival-disability paradox” presents heightened demands for rehabilitation medicine systems [18–20]. Leveraging the multidimensional analysis capabilities of the GBD database, this study provides a diachronic comprehensive view for assessing the burden of alcohol-related stroke. However, it is constrained by its ecological research design and does not thoroughly explore the socioeconomic determinants underlying regional disparities. Future prevention and control strategies should maintain the effectiveness of mortality control while focusing on developing a multidimensional system. This system should encompass the development of neuroprotective agents, optimization of community rehabilitation networks, and precise alcohol abstinence interventions. Particular emphasis should be placed on addressing early-onset stroke disability caused by alcohol abuse in young and middle-aged populations. In addition, the burden of alcohol-related stroke in high SDI regions improved most significantly (ASMR decreased by 61.7%, EAPC = −3.28), likely driven by the establishment of a comprehensive intervention system. First, the widespread implementation of early screening programs has substantially enhanced the ability to identify stroke risks. high SDI regions have successfully achieved early intervention for high-risk populations through universal health coverage policies. Second, the extensive use of statins has played a critical role in reducing YLLs (EAPC = −3.34) [21]. Systematic reviews indicate that statins effectively reduce stroke recurrence and short-term mortality by improving endothelial function, enhancing cerebral blood flow perfusion, and reducing infarct size, particularly when administered before or during the acute phase of stroke [22,23].

Over the past three decades, global alcohol-related stroke has exhibited a significant decline in ASMR, ASDR, and age-standardized rate of YLLs, indicating the substantial effectiveness of public health interventions focused on alcohol control policies and stroke emergency systems [24,25]. However, in stark contrast, the improvement in age-standardized rate of YLDs remains extremely limited. This “imbalance in mortality-disability burden” phenomenon suggests structural defects in the current prevention and control system. From a medical resource allocation perspective, global stroke intervention policies prioritize acute mortality control. For instance, the widespread adoption of thrombolytic thrombectomy technology has increased stroke survival rates [26–28], but a significant gap persists in long-term rehabilitation support for survivors (such as cognitive and motor function reconstruction) [29–31]. Research indicates that less than 20% of stroke patients in low-income countries receive standardized rehabilitation, and even in high-income countries, the coverage of multidisciplinary care in the chronic phase is below 60% [32–38]. This resource allocation model of “prioritizing rescue over rehabilitation” directly contributes to the accumulation of disabling sequelae, particularly limb dysfunction and cognitive impairment.

Gender and age differences reveal significant social determinants in the burden of alcohol-related stroke. Although the ASMR remains consistently higher for males, the decline in female mortality lags considerably (55.86% in males vs. 34.25% in females). This disparity may be attributed to the global increase in female alcohol consumption and sexual dimorphism in ethanol metabolism [39–43]. Females exhibit lower alcohol dehydrogenase activity compared to males [44], resulting in higher blood ethanol concentrations and more severe oxidative stress damage at equivalent consumption levels. Furthermore, the peak of male mortality and disability occurs 15–20 years earlier than in females (70–74 years for males vs. 85–89 years for females). This variance may be associated with high-risk drinking behaviors prevalent among young males, such as binge drinking and competitive consumption [45]. The age-standardized rate of YLDs for females continue to increase with age, highlighting systemic challenges in comorbidity management among the elderly population. females over 85 years often contend with concurrent conditions like hypertension and diabetes. The interaction of multiple diseases precipitates a marked decline in neural repair capacity post-stroke [46,47]. In the context of an aging population, this phenomenon may engender a “snowball effect” in the burden of stroke-related disability.

The emergence of high-burden areas results from the interaction between policy implementation and cultural risk factors. Eastern Europe exemplifies this phenomenon, where despite an annual per capita alcohol consumption of 15 liters (triple the WHO warning threshold) [48], alcohol tax comprises less than 20% of the retail price, significantly lower than the 60%−80% observed in Western European countries [49]. This policy gap has facilitated the widespread availability of inexpensive, high-alcohol content beverages, directly elevating the risk of Intracerebral hemorrhage. In Southeast Asia, cultural traditions influence strong alcohol consumption patterns. For instance, in Vietnam and neighboring countries, rice wine and herbal wine constitute 78% of total alcohol intake [50]. These beverages often contain excessive methanol impurities, inducing endothelial oxidative stress damage. Notably, sub-Saharan Africa, a medium- and low-SDI region, exhibits a lower burden potentially attributable to the prevalence of beer consumption (ethanol concentration <5%) and Islamic cultural prohibitions [50]. This observation suggests that alcohol type and religious norms play a regulatory role in shaping the disease spectrum.

The efficacy of alcohol control measures in high SDI regions underscores the significance of institutional intervention. Japan’s implementation of the “Alcohol Harm Prevention Law,” which mandates health warnings on alcohol advertisements, has resulted in a 41% reduction in drinking rates among working-age individuals over a decade [51,52]. Similarly, Singapore has employed pricing strategies, imposing a consumption tax of 75% on strong alcohol, effectively curbing high-risk drinking behavior. Conversely, in low- and medium-SDI regions, cultural transitions during economic development have exacerbated disease risks. Vietnam’s industrialization process exemplifies this phenomenon, where traditional community drinking practices (such as consuming strong alcohol communally in the “Solidarity Cup”) combined with commercial alcohol proliferation have led to a 230% increase in female drinking rates over ten years [53]. This paradigm of “cultural inertia coupled with market expansion” presents unique challenges for public health interventions.

Regional disparities in medical resource allocation further exacerbate the differentiation of disease burden. While East Asian countries demonstrate high stroke emergency coverage (with thrombolysis accessibility in China’s tertiary hospitals reaching 89%), the gap in rehabilitation services has led to a 30-day readmission rate of 22% among survivors [54]. This results in a decrease in age-standardized rate of YLLs but high age-standardized rate of YLDs. Conversely, in sub-Saharan Africa, the weak emergency system (with stroke unit coverage rate <5%) leads to numerous untreated patient deaths, creating a “statistical illusion” of low YLDs [55–57]. These findings underscore the necessity for a precise assessment framework: regions with robust emergency systems should prioritize the development of community rehabilitation networks, while areas with limited medical resources should focus on promoting low-cost stroke screening tools, such as portable carotid ultrasound.

The SII for overall stroke burden reversed from 52.31 in 1990 to −43.36 in 2021, indicating a shift in DALYs from higher socioeconomic status groups to lower-status groups. This trend aligns with the decline in the CII from 0.08 to −0.10, reflecting an increasing concentration of stroke burden among socioeconomically disadvantaged populations. A similar phenomenon is observed in cross-national generalization studies of neuroimaging diagnostic models, where underrepresentation of data from low-income countries may obscure true disparities in disease distribution [58]. For ischemic stroke, the SII decreased from 49.47 to −0.97, and the CII declined from 0.24 to 0.01, suggesting a significant reduction in the socioeconomic gradient. This improvement may be attributed to the widespread adoption of thrombolytic therapy and secondary prevention strategies; however, caution is warranted as “averaging” effects could mask the presence of high-risk subgroups [59,60]. In contrast, the SII and CII for intracerebral hemorrhage worsened to −47.40 and −0.19, respectively, with the absolute burden increasing ninefold. This reflects compounded risks faced by low socioeconomic groups due to alcohol abuse and limited access to medical care [61]. These findings are consistent with global observations of uneven neurosurgical resource distribution, where intracerebral hemorrhage mortality rates in low-income countries are 3.8 times higher than in high-income countries [60,62].

Frontier analysis reveals that Vietnam (efficiency gap of 378.52), Bulgaria (369.06), and Laos (310.31) exhibit the largest avoidable DALYs gaps relative to their SDI levels, while Somalia and Mali approach theoretical optimal values near zero. This paradoxical pattern requires contextualization within development levels. High SDI regions, such as Denmark (efficiency gap of 21.88), achieve cost-effective outcomes through evidence-based interventions and integrated health systems. Conversely, despite establishing stroke center networks, countries like Vietnam experience low resource utilization due to delayed primary care referrals and insufficient technical accessibility [63]. The abnormally high burden in Eastern European countries, exemplified by Bulgaria (intracerebral hemorrhage DALYs exceeding theoretical values by 256.14), may stem from reduced public health investment during economic transitions combined with high-risk behaviors such as heavy alcohol consumption [64]. In contrast, the “high efficiency” observed in low-income countries like Somalia reflects passive adaptation constrained by developmental limitations, underscoring the need to combine frontier analysis with absolute burden assessments to avoid misinterpreting low baselines as effective management [64]. This finding resonates with methodological critiques of public health goal evaluations, highlighting how simple coverage indicators may overlook the nonlinear impact of development disparities on progress rates [64].

Globally, the net increase in stroke DALYs was 243,000 per 100,000 population, driven primarily by aging (92.5%) and population growth (149.3%), partially offset by a 141.8% reduction in age-specific incidence rates through epidemiological improvements. The “exhaustion of the demographic dividend” is particularly evident in high SDI regions, where optimized hypertension management and other interventions led to a 257,000 DALY reduction (466.6% benefit). Middle SDI regions, however, experienced a 1.71 million DALYs surge due to aging (61.7%) and lifestyle changes accompanying economic growth (65.7%) [65,66]. Notably, decomposition of intracerebral hemorrhage burden shows that high SDI regions achieved a 99,020 DALY reduction through risk control measures, whereas low SDI regions remain constrained by insufficient medical resources [61]. This “development paradox” aligns with cardiovascular disease economic burden studies, emphasizing the dual pressures faced by middle-income countries during epidemiological transitions—aging populations and rising risk factors [67].

Research demonstrates that alcohol taxation is one of the most cost-effective intervention measures but exhibits significant regional economic dependencies in its effectiveness. The 2017 reform in Canada revealed that setting a minimum price of CAD 1.75 per standard drink reduced alcohol consumption by 8.68% and prevented 8,329 alcohol-related hospitalizations [68]. However, policy design must be carefully tailored to local economic structures. Denmark’s experience illustrates that phased tax increases (e.g., 20% or 100%) can mitigate the impact on low-income groups while supporting rehabilitation programs through tax redistribution, thereby achieving net cost savings [69]. For resource-constrained regions, implementing a “tiered tax rate model” is recommended—this involves setting differentiated tax baselines based on per capita GDP and directing revenue toward community rehabilitation network development to counteract resistance arising from economic inequality. Nevertheless, regional specificities pose multiple challenges. In sub-Saharan Africa, where illegal home-brewing accounts for up to 50% of alcohol consumption (e.g., Kenya), simply raising prices may expand the black market [70]. To address this, regulatory systems should be strengthened (e.g., deploying QR code traceability technology) alongside alternative livelihood support (e.g., Nigeria’s multi-sector employment program) [71]. Public awareness campaigns should also clarify how tax revenue contributes to healthcare improvements, enhancing public acceptance. Furthermore, the Ontario Stroke Rehabilitation Team in Canada achieved a 30% reduction in hospitalization rates through home visits and multidisciplinary collaboration, reducing per capita costs by 42% [72]. Similarly, Malaysia’s SCORE project utilized social organization networks to provide services at 10% lower costs than hospitals. These examples support the promotion of the “1+N” model (regional center professional training and grassroots site task transfer) [73]. However, policy implementation often faces constraints due to interdepartmental fragmentation and structural barriers. For instance, the disconnect between health policies and other social policies (e.g., employment, education) in Nigeria, as well as policy failure in Kenya’s Teso South region due to administrative corruption, financial scarcity, and traditional brewing culture, underscores the need for comprehensive governance [71]. Drawing on the EU’s “mainstreaming” framework, alcohol control should be integrated into cross-departmental assessment indicators (e.g., transportation, labor) and incentivized via central-local joint funds to enhance local execution [74]. A dual-track strategy is also essential: employing technical measures (e.g., mobile payment tracking) to curb corruption and culturally sensitive designs (e.g., using local art in Chhattisgarh, India, to promote anti-alcohol messages, increasing acceptance by 60%) to overcome cultural resistance [75,76]. In regions with diverse disease burdens, alcohol control risks marginalization. Referencing the WHO’s “Best Buy” list, bundling alcohol taxes with malaria drug procurement budgets and strengthening cost-benefit arguments (e.g., every dollar invested in alcohol taxes reduces stroke expenditures by 3.2 dollars) can secure political support [71,77]. Gender-differentiated strategies should not be overlooked; for example, Kerala, India, leveraged women’s self-help groups to promote low-alcohol beverages, reducing household alcohol expenditures by 28% and enhancing women’s economic empowerment [78]. This highlights the importance of integrating alcohol control policies into broader frameworks of social empowerment beyond a singular disease focus.

This study revealed that despite the declining trend in ASMR and ASDR of alcohol-related stroke in ischemic stroke and Intracerebral hemorrhage subtypes, the disease burden of Intracerebral hemorrhage remained significantly concentrated in high-income Asia-Pacific and Eastern Europe. Moreover, the age-standardized rate of YLDs of both subtypes showed no improvement, indicating potential subtype-specific deficiencies in the current prevention and control system. Analysis of pathological mechanisms suggests that the East Asian population’s high frequency mutation of the ALDH2 gene, a key enzyme in alcohol metabolism (with a mutation rate of 30%−40% at the rs671 site) [79–81], leads to acetaldehyde accumulation and exacerbates oxidative damage to the vascular endothelium. This may explain the higher proportion of Intracerebral hemorrhage in this region compared to the global average of 35%. Furthermore, the synergistic effect of alcohol and hypertension significantly contributes to the development of cerebral hemorrhage. Among moderate and heavy drinkers (53% of the study population), the prevalence of systolic hypertension (≥140 mmHg) is four times that of abstainers [82,83], with heavy drinkers having a relative risk of cerebral hemorrhage 3.13 times higher than abstainers [84]. These findings suggest that a simple alcohol control strategy may have limited efficacy in areas with high hypertension prevalence, such as Eastern European males, where hypertension prevalence exceeds 60%.

The stagnation of age-standardized rate of YLDs reveals significant deficiencies in the neurorehabilitation system. Among patients with upper limb dysfunction following ischemic stroke, merely 12% received standardized rehabilitation treatment, while the intervention rate for cognitive impairment in cerebral hemorrhage survivors was below 8% [85,86]. This inadequate rehabilitation approach contradicts the biological characteristics of the chronic neuroplasticity window period. Animal studies have demonstrated that dendritic remodeling capacity diminishes by 70% within 6 months post-stroke; however, the current median duration for initiating community rehabilitation intervention extends to 9.2 months [87–89].

### Limitations and strengths

However, the GBD study exhibits limitations in data quality, classification methods, model construction, and causal inference, which may compromise the reliability and generalizability of its conclusions. Regarding data quality, substantial regional disparities exist. In low-income countries, constrained by limited medical resources, stroke underreporting is a significant issue. For example, only 6% of hospitals in sub-Saharan Africa have dedicated stroke units, and primary healthcare facilities often lack essential diagnostic tools such as CT and MRI scanners, leading to a lower stroke diagnosis rate [90,91]. This not only underestimates stroke mortality rates in low-income countries—data indicate these rates are three times higher than in high-income countries—but also complicates accurate assessment of alcohol exposure. In regions dominated by informal economies (e.g., where 60–90% of alcohol consumption occurs in unregulated markets in sub-Saharan Africa), reliance on tax and sales records for alcohol consumption data introduces inaccuracies [92,93]. Furthermore, there are marked differences in alcohol beverage consumption trends across countries. Between 1960 and 2015, beer consumption increased from 3 liters to 11 liters per capita, yet the GBD model fails to adequately account for the heterogeneous effects of different beverage types on stroke risk [94,95]. Concerning stroke subtype classification, existing methods like the TOAST system demonstrate insufficient accuracy, achieving only 61% consistency with the gold standard and exhibiting a sensitivity of merely 33% for large artery atherosclerosis. Such limitations can introduce biases into alcohol-attributable effect estimates [96]. Additionally, the ICD coding system employed by GBD struggles to differentiate the pathophysiological mechanisms underlying stroke [97]. The alcohol consumption model suffers from temporal measurement biases and inadequately reflects changes in global alcohol consumption patterns from 1961 to 2014. In conflict-affected regions such as Sudan, flaws in the methodology for estimating per capita alcohol consumption result in outliers [92,94,95]. Moreover, ecological research methods are prone to ecological fallacy, time lag effects, and unmeasured confounding factors in causal inference. Future research could enhance the reliability and generalizability of GBD data through strategies such as establishing monitoring networks in low-income countries, integrating non-traditional data sources, developing subtype-specific attribution fractions, and conducting sensitivity analyses [91,92,95,98].

## Supporting information

S1 Table ASDR, Age-Standardized Rate of YLDs, Age-Standardized Rate of YLLs of high alcohol use-related Stroke between 1990 and 2021 at the global and regional level. (DOCX)

S2 Table ASMR, ASDR, Age-Standardized Rate of YLDs, and Age-Standardized Rate of YLLs of high alcohol use-related Stroke in 2021 at 204 countries and regions worldwide. (DOCX)

S3 Table ASMR, ASDR, Age-Standardized Rate of YLDs, and Age-Standardized Rate of YLLs of high alcohol use-related Stroke in males and females across 21 GBD regions and SDI regions in 2021. (DOCX)

S4 Table The number of Death, DALYs, YLDs, and YLLs of high alcohol use-related Stroke in different age groups in 2021. (DOCX)

S5 Table The rate of Death, DALYs, YLDs, and YLLs of high alcohol use-related Stroke in different age groups in 2021. (DOCX)

S6 Table ASMR for two types of high alcohol use-related Stroke in both sexes combined globally, 1990–2021. (DOCX)

S7 Table ASDR for two types of high alcohol use-related Stroke in both sexes combined globally, 1990–2021. (DOCX)

S8 Table Age-Standardized Rate of YLDs for two types of high alcohol use-related Stroke in both sexes combined globally, 1990–2021. (DOCX)

S9 Table Age-Standardized Rate of YLLs for two types of high alcohol use-related Stroke in both sexes combined globally, 1990–2021. (DOCX)

S1 Fig The EAPC of ASMR (A), ASDR (B), Age-Standardized Rate of YLDs (C), and Age-Standardized Rate of YLLs (D) of high alcohol use-related Stroke in both sexes combined across 21 GBD regions and SDI regions, 1990–2021. (TIFF)

S2 Fig The heatmap of two types high alcohol use-related Stroke showing ASMR (A), ASDR (B), Age-Standardized Rate of YLDs (C), and Age-Standardized Rate of YLLs (D) across 21 GBD regions in 2021. (TIFF)
