# Supplementary material for: The global burden of stroke attributable to high alcohol use from 1990 to 2021: An analysis for the global burden of disease study 2021
Source: PLoS One. 2025 Jul 14;20(7):e0328135. doi: 10.1371/journal.pone.0328135 (PMC12258592; doi:10.1371/journal.pone.0328135)
Supplement: S1 Table — (DOCX) [file pone.0328135.s001.docx]

**S1 Table:** ASDR, Age-Standardized Rate of YLDs, Age-Standardized Rate of YLLs of high alcohol use-related Stroke between 1990 and 2021 at the global and regional level. ASDR, age-standardized rate of DALYs; YLDs, years lived with disability; YLLs, years of life lost; SDI, socio-demographic index; UI, uncertainty intervals; CI, confidence interval; EAPC, estimated annual percentage change.

|  | **1990** | | | | **2021** | | | | **EAPC 1990-2021** | | | |
| --- | --- | --- | --- | --- | --- | --- | --- | --- | --- | --- | --- | --- |
|  | **ASMR**  **per 100,000, N (95% UI)** | **ASDR**  **per 100,000, N (95% UI)** | **Age-Standardized Rate of YLDs**  **per 100,000, N (95% UI)** | **Age-Standardized Rate of YLLs**  **per 100,000, N (95% UI)** | **ASMR**  **per 100,000, N (95% UI)** | **ASDR**  **per 100,000, N (95% UI)** | **Age-Standardized Rate of YLDs**  **per 100,000, N (95% UI)** | **Age-Standardized Rate of YLLs**  **per 100,000, N (95% UI)** | **ASMR**  **(95% CI)** | **ASDR**  **(95% CI)** | **Age-Standardized Rate of YLDs**  **(95% CI)** | **Age-Standardized Rate of YLLs**  **(95% CI)** |
| **Global** | 7.20(1.40,14.66) | 154.83(33.98,299.48) | 10.20(0.89-23.02) | 144.63(31.98-275.44) | 4.30(1.00,8.39) | 97.89(23.83,187.71) | 9.45(0.85-21.18) | 88.44(22.34-167.25) | -1.81(-1.88,-1.75) | -1.63(-1.70,-1.56) | -0.25(-0.29,-0.20) | -1.75(-1.82,-1.67) |
| Male | 12.38(2.55,24.70) | 265.85(59.02,505.77) | 15.92(1.51-35.61) | 249.93(57.41-469.43) | 8.14(1.91,15.74) | 181.55(44.50,345.60) | 3.76(0.22-9.07) | 165.61(42.25-311.59) | -1.47(-1.53,-1.41) | -1.34(-1.40,-1.28) | 0.04(-0.02,0.10) | -1.45(-1.52,-1.38) |
| Female | 3.35(0.46,7.25) | 62.51(10.14,131.75) | 5.41(0.35-13.03) | 57.10(9.72-119.05) | 1.22(0.20,2.59) | 24.89(4.72,51.84) | 9.45(0.85-21.18) | 21.12(4.45-43.08) | -3.53(-3.66,-3.39) | -3.30(-3.44,-3.16) | -1.28(-1.31,-1.25) | -3.56(-3.72,-3.40) |
| **SDI** |  |  |  |  |  |  |  |  |  |  |  |  |
| High SDI | 6.50(0.91,13.60) | 132.55(22.99-266.22) | 14.76(1.10-34.59) | 117.78(21.66-236.66) | 2.49(0.45,5.05) | 57.25(10.71-115.15) | 12.83(0.65-29.37) | 44.42(9.23-86.29) | -3.28(-3.40,-3.17) | -2.88(-2.99--2.77) | -0.52(-0.56,-0.47) | -3.34(-3.46,-3.22) |
| High-middle SDI | 11.56(2.06,23.67) | 250.70(51.62-489.75) | 14.55(1.33-32.68) | 236.14(50.08-456.45) | 6.03(1.19,12.28) | 134.48(30.10-266.02) | 12.70(0.98-28.60) | 121.78(29.66-239.58) | -2.45(-2.69,-2.22) | -2.39(-2.63--2.14) | -0.49(-0.54,-0.44) | -2.53(-2.80,-2.26) |
| Middle SDI | 5.97(1.39,11.48) | 141.18(31.07-275.13) | 6.29(0.80-13.65) | 134.89(29.06-263.18) | 5.24(1.28,9.89) | 118.52(30.96-219.97) | 8.34(0.86-18.23) | 110.18(28.18-203.71) | -0.33(-0.44,-0.22) | -0.45(-0.55--0.34) | 1.06(0.90,1.21) | -0.53(-0.64,-0.43) |
| Low-middle SDI | 2.36(0.57,4.74) | 58.03(12.79-114.64) | 2.67(0.33-6.06) | 55.36(11.92-109.29) | 2.56(0.63,5.02) | 64.04(14.92-123.09) | 3.51(0.49-7.79) | 60.52(14.02-116.94) | 0.37(0.28,0.46) | 0.43(0.34-0.52) | 1.06(0.97,1.16) | 0.40(0.31,0.49) |
| Low SDI | 3.84(0.79,7.42) | 91.01(17.08-181.35) | 3.69(0.29-8.59) | 87.32(16.21-174.96) | 3.38(0.82,6.46) | 79.17(18.21-153.35) | 4.25(0.43-9.65) | 74.92(16.57-146.48) | -0.51(-0.76,-0.26) | -0.58(-0.83--0.33) | 0.50(0.28,0.72) | -0.63(-0.88,-0.38) |
| **GBD region** |  |  |  |  |  |  |  |  |  |  |  |  |
| Andean Latin America | 3.03(0.67,5.94) | 73.93(14.09-145.26) | 3.13(0.27-7.34) | 70.80(13.95-138.47) | 1.63(0.35,3.27) | 39.85(8.71-77.75) | 2.87(0.21-6.57) | 36.98(7.78-72.71) | -1.97(-2.29,-1.66) | -1.99(-2.31--1.66) | -0.11(-0.27,0.06) | -2.10(-2.44,-1.76) |
| Australasia | 5.61(0.53,13.92) | 101.00(14.31-238.12) | 12.28(0.30-32.73) | 88.72(13.48-208.66) | 2.17(0.35,4.46) | 41.63(6.73-84.85) | 10.44(-0.11-24.59) | 31.18(6.08-61.32) | -3.12(-3.19,-3.06) | -2.93(-3.01--2.85) | -0.53(-0.59,-0.47) | -3.47(-3.55,-3.38) |
| Caribbean | 4.23(0.98,8.62) | 107.37(23.34-213.11) | 3.43(0.44-7.88) | 103.94(22.15-205.93) | 3.20(0.75,6.41) | 81.18(17.86-164.74) | 3.38(0.36-7.93) | 77.80(16.67-157.41) | -0.80(-0.88,-0.73) | -0.80(-0.91--0.70) | -0.03(-0.09,0.04) | -0.83(-0.94,-0.72) |
| Central Asia | 5.77(1.10,12.38) | 160.00(35.99-326.55) | 12.25(1.38-28.68) | 147.75(33.74-299.92) | 4.74(0.83,10.71) | 120.51(24.14-251.92) | 10.36(0.98-25.76) | 110.16(23.15-231.49) | -0.92(-1.25,-0.60) | -1.34(-1.70--0.97) | -0.64(-0.68,-0.59) | -1.39(-1.78,-1.00) |
| Central Europe | 16.26(2.20,34.13) | 335.97(59.13-669.09) | 19.55(1.19-43.79) | 316.42(57.23-629.75) | 7.45(1.10,15.70) | 155.50(25.41-311.62) | 15.35(-0.02-35.68) | 140.16(23.15-278.12) | -2.93(-3.11,-2.75) | -2.94(-3.13--2.75) | -0.89(-0.95,-0.83) | -3.10(-3.30,-2.90) |
| Central Latin America | 2.50(0.53,5.15) | 57.83(13.15-111.36) | 3.18(0.27-7.75) | 54.66(12.63-105.52) | 1.18(0.26,2.41) | 29.17(6.69-57.80) | 2.18(0.18-5.44) | 26.99(6.47-53.91) | -2.91(-3.15,-2.67) | -2.76(-3.02--2.49) | -1.51(-1.67,-1.35) | -2.84(-3.12,-2.57) |
| Central Sub-Saharan Africa | 6.58(1.20,13.30) | 144.27(25.44-290.94) | 6.37(0.37-15.51) | 137.91(24.43-275.55) | 5.53(1.07,11.37) | 122.94(21.82-253.35) | 6.15(0.47-15.04) | 116.79(21.04-240.22) | -0.29(-0.96,0.39) | -0.21(-0.88-0.46) | 0.36(-0.34,1.08) | -0.24(-0.91,0.43) |
| East Asia | 10.59(2.47,20.32) | 239.32(51.99-459.13) | 10.67(1.52-22.51) | 228.65(49.66-440.57) | 7.79(1.83,14.61) | 170.56(42.18-320.85) | 13.96(1.34-30.70) | 156.61(40.16-295.44) | -0.92(-1.04,-0.79) | -1.01(-1.11--0.90) | 1.03(0.81,1.25) | -1.14(-1.25,-1.02) |
| Eastern Europe | 11.39(1.06,27.14) | 269.63(35.17-595.23) | 17.46(0.93-41.70) | 252.18(33.80-552.34) | 6.63(0.77,16.04) | 170.28(26.94-379.42) | 15.10(0.44-38.29) | 155.18(26.47-345.44) | -2.60(-3.29,-1.91) | -2.34(-3.04--1.63) | -0.51(-0.75,-0.27) | -2.47(-3.20,-1.73) |
| Eastern Sub-Saharan Africa | 5.94(1.07,11.95) | 140.11(21.86-288.47) | 5.73(0.32-13.41) | 134.38(20.29-276.62) | 4.93(1.11,9.54) | 114.78(24.60-224.38) | 6.52(0.50-15.03) | 108.26(22.78-212.45) | -0.83(-1.04,-0.62) | -0.91(-1.13--0.69) | 0.43(0.24,0.62) | -0.98(-1.20,-0.75) |
| High-income Asia Pacific | 8.10(1.53,15.76) | 172.46(34.61-319.25) | 22.05(2.49-47.93) | 150.41(31.66-276.74) | 2.16(0.44,4.19) | 54.96(10.14-104.91) | 14.80(1.45-32.48) | 40.16(8.33-76.26) | -4.63(-4.81,-4.45) | -4.03(-4.21--3.85) | -1.59(-1.73,-1.44) | -4.61(-4.79,-4.43) |
| High-income North America | 2.00(0.31,5.27) | 48.37(9.21-118.57) | 8.93(0.33-24.88) | 39.45(8.01-94.88) | 1.92(0.35,4.13) | 47.33(9.23-98.08) | 12.22(0.51-29.57) | 35.12(8.34-68.72) | -0.25(-0.39,-0.10) | -0.15(-0.24--0.05) | 1.08(0.89,1.27) | -0.51(-0.65,-0.36) |
| North Africa and Middle East | 0.76(0.14,1.64) | 19.11(4.15-40.57) | 1.17(0.13-2.98) | 17.94(3.76-37.73) | 0.37(0.05,0.85) | 8.65(1.51-19.41) | 0.89(0.06-2.44) | 7.76(1.41-17.24) | -2.58(-2.71,-2.45) | -2.83(-2.94--2.72) | -1.13(-1.25,-1.01) | -2.98(-3.10,-2.86) |
| Oceania | 2.69(0.39,6.09) | 72.30(10.01-161.07) | 3.39(0.47-7.83) | 68.91(8.73-155.73) | 1.92(0.30,4.25) | 51.18(7.09-113.92) | 2.97(0.41-7.02) | 48.21(6.46-108.53) | -0.87(-1.22,-0.52) | -0.90(-1.24--0.56) | -0.25(-0.43,-0.06) | -0.93(-1.28,-0.59) |
| South Asia | 1.48(0.25,3.26) | 38.01(6.43-81.45) | 1.79(0.23-4.32) | 36.21(6.03-77.14) | 1.84(0.43,3.82) | 46.10(11.19-93.57) | 2.75(0.43-6.35) | 43.35(10.19-88.79) | 0.92(0.68,1.16) | 0.83(0.59-1.07) | 1.71(1.53,1.89) | 0.78(0.54,1.02) |
| Southeast Asia | 2.40(0.55,4.57) | 61.86(12.27-120.77) | 3.68(0.35-8.21) | 58.18(11.60-112.56) | 5.08(1.25,9.30) | 126.87(31.10-236.57) | 7.59(1.06-15.78) | 119.28(29.06-222.98) | 2.86(2.54,3.17) | 2.73(2.44-3.02) | 2.60(2.42,2.78) | 2.74(2.44,3.04) |
| Southern Latin America | 10.38(2.30,19.87) | 236.18(60.09-432.76) | 18.43(2.44-38.08) | 217.75(58.25-398.56) | 3.13(0.63,6.13) | 70.72(15.93-135.18) | 10.11(0.90-22.79) | 60.61(14.26-113.08) | -3.57(-3.73,-3.40) | -3.73(-3.88--3.57) | -2.00(-2.13,-1.87) | -3.93(-4.09,-3.77) |
| Southern Sub-Saharan Africa | 5.71(1.28,11.11) | 138.09(30.28-268.62) | 11.60(0.94-26.94) | 126.49(27.96-247.12) | 5.73(1.39,11.31) | 131.41(31.44-253.37) | 8.59(0.65-20.74) | 122.81(28.38-236.57) | -0.12(-0.57,0.33) | -0.31(-0.72-0.09) | -1.28(-1.39,-1.17) | -0.24(-0.67,0.20) |
| Tropical Latin America | 6.24(1.22,12.20) | 152.88(36.15-289.35) | 4.80(0.49-10.96) | 148.09(34.59-277.87) | 2.48(0.49,4.93) | 58.04(12.81-109.68) | 3.32(0.25-7.87) | 54.72(12.44-104.58) | -3.02(-3.16,-2.88) | -3.28(-3.40--3.16) | -1.44(-1.61,-1.27) | -3.37(-3.49,-3.24) |
| Western Europe | 8.90(1.08,18.76) | 159.91(24.66-326.36) | 14.96(0.61-34.88) | 144.95(23.40-292.26) | 2.59(0.47,5.20) | 51.65(9.34-102.07) | 11.29(0.24-25.93) | 40.36(8.19-77.45) | -4.12(-4.25,-3.99) | -3.80(-3.94--3.66) | -0.95(-0.99,-0.91) | -4.30(-4.44,-4.15) |
| Western Sub-Saharan Africa | 7.29(1.62,14.51) | 164.15(37.97-320.15) | 8.21(0.91-18.62) | 155.94(36.43-301.42) | 6.48(1.28,12.79) | 143.94(31.56-277.83) | 9.09(0.87-20.46) | 134.85(30.73-259.67) | -0.53(-0.67,-0.39) | -0.58(-0.74--0.42) | 0.24(0.17,0.32) | -0.63(-0.79,-0.47) |
