# Supplementary material for: The global burden of stroke attributable to high alcohol use from 1990 to 2021: An analysis for the global burden of disease study 2021
Source: PLoS One. 2025 Jul 14;20(7):e0328135. doi: 10.1371/journal.pone.0328135 (PMC12258592; doi:10.1371/journal.pone.0328135)
Supplement: S2 Table — (DOCX) [file pone.0328135.s002.docx]

**S2 Table:** ASMR, ASDR, Age-Standardized Rate of YLDs, and Age-Standardized Rate of YLLs of high alcohol use-related Stroke in 2021 at 204 countries and regions worldwide. ASMR, age-standardized mortality rate; DALYs, disability-adjusted life years; ASDR, age-standardized rate of DALYs; YLDs, years lived with disability; YLLs, years of life lost.

| **Location Name** | **ASMR**  **per 100,000, N (95% UI)** | **ASDR**  **per 100,000, N (95% UI)** | **Age-Standardized Rate of YLDs**  **per 100,000, N (95% UI)** | **Age-Standardized Rate of YLLs**  **per 100,000, N (95% UI)** |
| --- | --- | --- | --- | --- |
| Democratic People's Republic of Korea | 7.69(1.81-14.25) | 211.11(47.73-392.04) | 11.57(1.37-25.06) | 199.54(43.68-376.49) |
| China | 7.94(1.85-14.91) | 172.51(42.43-325.53) | 14.08(1.35-30.94) | 158.43(40.39-298.59) |
| Taiwan (Province of China) | 1.47(0.34-2.84) | 45.04(10.80-85.39) | 9.15(0.98-20.54) | 35.89(7.39-70.08) |
| Cambodia | 8.43(1.83-15.77) | 201.35(39.24-379.07) | 10.93(1.66-23.42) | 190.41(36.96-360.01) |
| Indonesia | 0.65(0.12-1.49) | 17.18(2.98-39.64) | 1.04(0.11-2.80) | 16.14(2.76-37.63) |
| Lao People's Democratic Republic | 12.21(2.92-22.91) | 310.31(77.64-589.73) | 17.18(2.42-38.20) | 293.13(69.63-562.12) |
| Maldives | 0.62(0.09-1.44) | 15.58(1.70-36.71) | 1.94(0.07-5.07) | 13.64(1.58-32.45) |
| Philippines | 8.02(2.03-14.79) | 221.97(52.41-409.01) | 14.49(2.19-30.64) | 207.48(46.34-383.33) |
| Thailand | 2.85(0.70-5.65) | 87.18(19.97-166.79) | 8.68(1.23-18.24) | 78.50(16.69-154.03) |
| Myanmar | 5.88(1.30-11.68) | 156.74(33.04-313.31) | 7.04(1.09-14.79) | 149.70(29.76-300.58) |
| Timor-Leste | 5.28(1.23-10.62) | 129.67(28.86-263.35) | 6.26(0.88-13.56) | 123.41(27.96-253.27) |
| Malaysia | 1.08(0.24-2.31) | 29.78(6.29-64.02) | 3.33(0.34-8.26) | 26.46(5.82-56.76) |
| Sri Lanka | 2.73(0.56-6.11) | 60.16(12.97-126.34) | 5.06(0.50-11.86) | 55.10(11.56-116.14) |
| Viet Nam | 16.22(4.02-29.11) | 378.50(84.09-688.25) | 18.15(2.35-36.78) | 360.35(82.87-660.63) |
| Fiji | 2.56(0.45-5.47) | 69.60(12.17-149.30) | 5.84(0.72-13.65) | 63.76(9.29-139.71) |
| Marshall Islands | 4.08(0.56-9.36) | 115.34(12.91-266.67) | 5.59(0.89-12.95) | 109.75(10.41-254.97) |
| Kiribati | 1.45(0.06-4.44) | 44.78(-0.26-139.07) | 3.06(0.04-9.27) | 41.72(-0.32-131.17) |
| Samoa | 2.56(0.36-5.90) | 71.88(11.33-162.53) | 5.04(0.66-11.64) | 66.84(9.33-153.56) |
| Micronesia (Federated States of) | 4.09(0.61-9.27) | 121.28(14.70-277.47) | 6.38(0.98-14.94) | 114.90(12.42-262.41) |
| Papua New Guinea | 1.72(0.23-3.98) | 44.33(5.76-104.07) | 1.96(0.27-4.68) | 42.37(5.12-101.19) |
| Solomon Islands | 1.95(0.23-4.63) | 55.71(5.40-135.29) | 3.32(0.45-7.79) | 52.39(4.61-128.26) |
| Vanuatu | 3.76(0.57-8.30) | 109.29(14.72-242.41) | 6.85(1.08-15.58) | 102.44(12.93-230.34) |
| Tonga | 0.60(0.08-1.46) | 15.97(2.07-38.59) | 1.71(0.11-4.46) | 14.26(1.87-35.42) |
| Poland | 4.72(0.62-10.28) | 105.47(16.57-217.67) | 11.76(0.17-27.74) | 93.71(15.01-192.14) |
| Azerbaijan | 3.53(0.57-8.19) | 85.03(16.55-187.74) | 7.77(0.94-19.45) | 77.26(14.58-172.48) |
| Mongolia | 7.64(1.00-16.16) | 210.40(27.51-429.36) | 15.09(2.59-33.70) | 195.30(24.32-403.18) |
| Singapore | 0.25(0.04-0.49) | 9.28(1.40-18.92) | 3.69(0.24-8.70) | 5.59(0.77-11.58) |
| Kazakhstan | 7.17(1.19-17.36) | 174.41(34.01-380.04) | 15.54(1.02-39.91) | 158.86(31.71-347.14) |
| Georgia | 8.34(1.44-18.84) | 193.02(40.67-409.91) | 12.97(1.47-29.61) | 180.05(36.71-383.62) |
| Australia | 2.07(0.33-4.20) | 40.49(6.77-81.85) | 10.64(-0.09-24.77) | 29.86(6.06-58.07) |
| Tajikistan | 0.93(0.18-2.06) | 30.40(6.12-64.26) | 2.60(0.29-6.39) | 27.80(5.57-58.96) |
| Armenia | 2.12(0.21-5.34) | 56.22(6.70-130.99) | 7.62(-0.02-19.99) | 48.60(6.71-110.59) |
| Serbia | 10.18(1.15-23.54) | 194.20(28.66-420.56) | 13.19(-0.08-31.07) | 181.01(26.49-390.18) |
| Kyrgyzstan | 3.29(0.52-7.70) | 98.23(16.95-217.64) | 7.24(0.73-17.40) | 90.99(16.05-198.89) |
| Belarus | 7.14(0.73-16.79) | 182.69(26.12-394.84) | 18.98(0.41-46.39) | 163.72(24.75-352.74) |
| Estonia | 2.71(0.24-6.69) | 69.67(8.56-158.17) | 12.84(-0.07-32.77) | 56.83(8.09-125.06) |
| France | 2.29(0.42-4.53) | 46.53(8.53-90.63) | 11.21(0.24-25.33) | 35.32(7.18-67.14) |
| Ukraine | 6.03(0.83-15.56) | 156.44(27.52-369.80) | 15.56(0.44-40.34) | 140.88(25.21-330.64) |
| Japan | 2.10(0.43-4.04) | 54.28(10.27-101.59) | 14.72(1.35-32.76) | 39.56(8.70-72.10) |
| Albania | 6.95(1.16-14.59) | 119.25(24.35-244.24) | 7.21(0.65-16.48) | 112.03(22.70-230.70) |
| Russian Federation | 6.80(0.75-16.01) | 174.87(26.45-380.19) | 14.49(0.49-36.89) | 160.38(26.37-343.89) |
| Uzbekistan | 3.22(0.58-7.23) | 89.46(17.21-191.69) | 9.12(0.88-22.00) | 80.34(16.28-171.50) |
| Brunei Darussalam | 0.19(0.01-0.46) | 4.85(0.06-11.74) | 0.80(-0.01-2.29) | 4.05(0.05-9.72) |
| Croatia | 5.47(0.74-11.81) | 107.48(16.75-217.55) | 14.56(-0.55-34.12) | 92.92(16.49-187.76) |
| Andorra | 1.91(0.25-3.89) | 37.12(5.40-72.82) | 8.39(0.40-19.12) | 28.73(4.85-54.95) |
| Belgium | 2.61(0.51-5.12) | 51.07(9.20-98.77) | 9.73(0.11-22.22) | 41.34(8.48-77.33) |
| Bulgaria | 18.16(2.30-38.39) | 369.04(58.16-731.32) | 23.68(0.42-53.44) | 345.36(56.88-680.23) |
| Turkmenistan | 7.57(1.23-16.63) | 211.69(38.71-434.22) | 13.66(1.29-33.09) | 198.02(37.40-411.60) |
| Montenegro | 15.19(2.94-30.10) | 266.22(54.18-518.53) | 11.72(1.30-25.77) | 254.50(50.87-498.39) |
| Bosnia and Herzegovina | 6.41(0.49-13.97) | 133.09(12.81-287.38) | 15.11(-1.02-35.47) | 117.98(13.02-251.85) |
| Finland | 2.31(0.38-4.72) | 50.34(8.33-102.47) | 12.75(0.67-28.50) | 37.59(6.65-73.60) |
| Italy | 2.90(0.60-5.71) | 50.28(10.22-97.56) | 7.32(0.53-16.55) | 42.96(8.81-81.02) |
| Czechia | 4.32(0.48-9.22) | 92.65(12.36-189.39) | 19.10(-0.40-44.32) | 73.54(11.53-150.90) |
| Greece | 3.88(0.70-7.56) | 73.37(15.80-138.73) | 9.39(0.78-21.12) | 63.99(14.63-121.16) |
| North Macedonia | 17.50(1.74-38.61) | 301.81(43.13-623.58) | 16.80(0.23-37.82) | 285.02(39.83-584.85) |
| Germany | 3.07(0.46-6.15) | 67.40(10.09-135.92) | 17.70(-0.18-40.37) | 49.69(9.25-96.92) |
| Hungary | 4.96(0.51-10.67) | 113.39(15.29-235.50) | 14.67(-0.34-33.74) | 98.73(15.99-199.81) |
| Netherlands | 3.04(0.44-6.18) | 53.62(8.64-108.80) | 10.61(0.14-24.49) | 43.01(8.23-86.09) |
| Norway | 1.75(0.30-3.63) | 36.33(5.86-75.67) | 10.80(0.04-25.87) | 25.53(4.96-50.91) |
| Cyprus | 3.15(0.44-6.60) | 49.09(9.48-96.42) | 4.84(0.61-10.44) | 44.25(8.66-86.44) |
| Romania | 11.49(1.65-24.07) | 235.35(44.39-466.97) | 18.77(0.07-43.45) | 216.58(43.08-431.60) |
| Slovakia | 6.44(0.80-13.93) | 143.01(21.20-296.15) | 21.44(-1.02-49.73) | 121.57(19.34-250.29) |
| Israel | 0.69(0.12-1.39) | 15.96(2.78-32.93) | 3.86(-0.11-9.87) | 12.10(2.38-24.20) |
| Slovenia | 2.23(0.16-5.54) | 42.01(3.70-102.87) | 7.00(-0.03-18.80) | 35.00(3.50-82.25) |
| Latvia | 8.38(0.64-21.43) | 184.89(21.79-425.51) | 20.34(-0.64-51.06) | 164.55(19.68-374.95) |
| Republic of Moldova | 7.66(1.15-16.70) | 193.54(35.76-401.05) | 16.72(0.91-41.23) | 176.81(33.03-360.40) |
| Lithuania | 5.33(0.44-13.40) | 127.05(14.74-289.83) | 17.87(-0.32-45.87) | 109.18(13.55-248.36) |
| Republic of Korea | 2.69(0.49-5.69) | 63.56(10.85-131.28) | 15.77(1.49-35.58) | 47.79(9.47-98.51) |
| New Zealand | 2.74(0.41-5.64) | 48.01(7.10-98.91) | 9.44(0.01-23.48) | 38.56(6.78-77.08) |
| Austria | 2.02(0.34-4.12) | 46.32(7.01-93.81) | 15.60(0.37-36.79) | 30.72(5.76-60.45) |
| Denmark | 3.28(0.56-6.58) | 59.36(10.62-117.33) | 10.25(0.19-23.35) | 49.11(9.76-96.14) |
| Portugal | 4.01(0.87-7.90) | 73.00(14.78-136.84) | 7.34(0.67-15.92) | 65.66(14.63-123.66) |
| Luxembourg | 2.88(0.55-5.64) | 51.28(10.51-98.33) | 7.81(0.55-17.25) | 43.47(8.97-82.11) |
| Iceland | 1.75(0.30-3.62) | 34.83(6.03-70.50) | 9.51(0.14-22.04) | 25.32(4.92-50.71) |
| Malta | 1.62(0.30-3.36) | 31.66(6.33-64.09) | 5.27(0.58-11.83) | 26.39(5.50-53.34) |
| Ireland | 1.77(0.25-3.67) | 32.28(5.54-66.55) | 6.68(0.57-15.38) | 25.61(4.90-52.03) |
| Spain | 1.87(0.35-3.63) | 41.00(7.92-79.66) | 9.90(0.11-23.15) | 31.09(7.19-58.14) |
| Switzerland | 1.73(0.27-3.50) | 32.84(5.15-65.46) | 8.51(0.29-19.12) | 24.33(4.71-47.25) |
| Sweden | 2.20(0.36-4.55) | 43.40(7.23-88.14) | 11.60(0.88-26.57) | 31.80(5.89-62.59) |
| United Kingdom | 2.32(0.42-4.62) | 45.35(8.17-89.83) | 9.84(0.17-22.51) | 35.51(7.12-68.73) |
| Argentina | 3.04(0.70-5.75) | 71.20(16.38-132.00) | 9.72(0.85-21.66) | 61.47(14.02-114.56) |
| United States of America | 1.99(0.37-4.26) | 48.87(9.61-101.19) | 12.19(0.49-29.66) | 36.68(8.68-71.88) |
| Canada | 1.35(0.22-2.96) | 34.60(5.91-73.86) | 12.42(0.54-29.78) | 22.18(4.54-45.17) |
| Antigua and Barbuda | 3.27(0.75-6.49) | 71.68(16.24-137.72) | 3.23(0.36-7.44) | 68.45(15.61-131.21) |
| Uruguay | 3.71(0.61-7.60) | 76.86(14.87-154.11) | 9.97(0.73-23.23) | 66.90(12.44-133.33) |
| Chile | 3.20(0.57-6.37) | 68.52(13.47-134.36) | 11.01(1.04-24.42) | 57.51(12.57-110.46) |
| Cuba | 2.46(0.45-5.21) | 56.97(12.05-115.47) | 3.15(0.26-7.52) | 53.82(12.06-108.17) |
| Dominican Republic | 3.27(0.71-6.69) | 87.30(20.12-174.13) | 4.17(0.46-9.58) | 83.13(18.31-167.56) |
| Bahamas | 2.19(0.41-4.44) | 56.37(10.68-111.05) | 2.99(0.33-6.96) | 53.38(9.45-105.99) |
| Belize | 2.08(0.52-4.16) | 52.27(11.71-103.46) | 2.58(0.34-5.74) | 49.69(11.15-98.60) |
| Grenada | 3.78(0.88-7.68) | 87.80(19.83-176.64) | 3.88(0.39-9.35) | 83.92(18.57-169.78) |
| Dominica | 4.06(0.94-8.29) | 93.69(21.12-185.46) | 3.18(0.31-7.39) | 90.51(20.49-178.84) |
| Guyana | 6.08(1.35-12.81) | 150.34(31.55-306.24) | 4.16(0.50-9.28) | 146.18(30.76-298.97) |
| Jamaica | 2.90(0.58-6.16) | 65.59(13.23-136.90) | 2.39(0.30-5.56) | 63.19(12.49-131.70) |
| Saint Lucia | 4.90(1.09-10.11) | 108.18(25.78-213.76) | 5.10(0.58-11.59) | 103.08(24.07-205.20) |
| Haiti | 8.00(1.79-17.32) | 184.68(40.99-398.60) | 3.80(0.49-8.55) | 180.88(39.96-390.58) |
| Trinidad and Tobago | 3.37(0.62-7.28) | 78.05(17.25-159.30) | 4.02(0.28-9.69) | 74.03(16.35-149.46) |
| Suriname | 3.85(0.91-7.92) | 100.35(22.85-196.68) | 3.37(0.43-7.72) | 96.98(21.65-190.78) |
| Barbados | 3.40(0.66-7.16) | 73.00(16.66-151.07) | 4.26(0.40-10.08) | 68.74(16.25-143.91) |
| Saint Vincent and the Grenadines | 5.79(1.27-11.38) | 131.34(31.91-254.79) | 4.98(0.52-10.91) | 126.37(30.89-247.66) |
| Bolivia (Plurinational State of) | 2.90(0.63-5.77) | 63.54(13.43-128.71) | 2.65(0.21-6.26) | 60.89(12.27-123.87) |
| Peru | 1.67(0.37-3.35) | 41.49(8.49-80.21) | 3.37(0.25-7.59) | 38.12(8.21-74.26) |
| El Salvador | 0.87(0.19-1.89) | 21.98(4.80-44.71) | 1.29(0.11-3.25) | 20.69(4.67-42.21) |
| Mexico | 1.22(0.25-2.55) | 30.35(6.25-59.81) | 2.64(0.21-6.55) | 27.71(6.04-54.89) |
| Costa Rica | 1.02(0.20-2.19) | 22.57(5.17-45.82) | 2.36(0.18-5.86) | 20.21(4.60-40.29) |
| Honduras | 2.59(0.57-5.37) | 56.71(12.13-114.43) | 1.42(0.12-3.48) | 55.29(11.84-112.50) |
| Nicaragua | 1.02(0.23-2.11) | 25.20(5.64-50.54) | 1.93(0.18-4.74) | 23.27(5.29-47.85) |
| Guatemala | 0.91(0.20-2.04) | 21.22(4.48-44.60) | 1.23(0.14-2.90) | 19.99(4.07-42.42) |
| Venezuela (Bolivarian Republic of) | 1.90(0.44-4.13) | 46.13(10.33-97.97) | 2.05(0.24-4.84) | 44.08(9.71-93.66) |
| Panama | 1.81(0.39-3.89) | 41.07(9.38-82.66) | 2.66(0.31-6.28) | 38.41(8.80-77.03) |
| Ecuador | 0.91(0.19-1.87) | 23.74(5.28-49.20) | 1.97(0.13-4.78) | 21.77(4.87-46.41) |
| Colombia | 0.66(0.15-1.49) | 16.48(3.64-35.29) | 1.52(0.12-3.80) | 14.95(3.26-32.52) |
| Paraguay | 5.05(1.15-10.51) | 109.46(24.26-223.36) | 5.07(0.39-11.58) | 104.39(23.00-213.59) |
| Algeria | 0.37(0.04-0.93) | 8.01(1.08-18.44) | 0.93(0.06-2.60) | 7.08(1.05-16.24) |
| Brazil | 2.42(0.47-4.80) | 56.87(12.36-107.73) | 3.28(0.24-7.80) | 53.59(12.00-102.41) |
| Iran (Islamic Republic of) | 0.25(0.02-0.65) | 6.42(0.71-15.84) | 0.76(0.03-2.18) | 5.66(0.73-13.76) |
| Jordan | 0.14(0.01-0.35) | 3.33(0.37-8.44) | 0.52(-0.00-1.57) | 2.81(0.37-6.66) |
| Morocco | 0.08(0.01-0.19) | 2.73(0.48-6.14) | 0.31(0.02-0.84) | 2.42(0.44-5.57) |
| Iraq | 0.26(0.04-0.63) | 5.99(1.07-14.26) | 0.34(0.03-0.90) | 5.65(1.02-13.47) |
| Libya | 0.17(0.02-0.44) | 4.35(0.66-10.63) | 0.39(0.02-1.12) | 3.96(0.63-9.60) |
| Kuwait | 0.02(0.00-0.07) | 0.56(-0.01-1.86) | 0.11(-0.00-0.41) | 0.45(-0.00-1.46) |
| Palestine | 0.51(0.07-1.28) | 11.85(1.86-28.69) | 0.80(0.06-2.20) | 11.05(1.76-26.75) |
| Bahrain | 0.30(0.04-0.69) | 6.99(1.31-15.96) | 0.70(0.07-1.79) | 6.28(1.18-14.12) |
| Egypt | 0.24(0.03-0.61) | 5.92(0.81-14.04) | 0.35(0.02-1.01) | 5.57(0.78-13.33) |
| Lebanon | 0.58(0.08-1.31) | 14.80(2.83-33.23) | 2.48(0.14-6.69) | 12.32(2.43-26.77) |
| Oman | 0.17(0.02-0.45) | 4.61(0.70-11.63) | 0.58(0.03-1.68) | 4.02(0.63-10.19) |
| Saudi Arabia | 0.11(0.01-0.32) | 3.03(0.23-8.65) | 0.20(0.01-0.64) | 2.83(0.21-7.98) |
| Qatar | 0.15(0.02-0.37) | 4.08(0.69-9.54) | 0.76(0.08-2.07) | 3.32(0.61-7.74) |
| Tunisia | 0.57(0.07-1.40) | 14.31(2.15-33.78) | 1.41(0.10-3.83) | 12.90(2.03-29.91) |
| United Arab Emirates | 1.12(0.11-2.79) | 24.57(2.90-60.97) | 4.39(0.09-12.52) | 20.18(2.68-50.18) |
| Syrian Arab Republic | 0.46(0.06-1.12) | 11.12(1.64-27.14) | 0.77(0.08-2.11) | 10.34(1.55-25.00) |
| Afghanistan | 0.09(0.01-0.25) | 2.47(0.25-6.36) | 0.11(0.00-0.36) | 2.36(0.24-6.12) |
| Türkiye | 0.74(0.12-1.69) | 17.81(3.78-39.00) | 2.03(0.19-5.32) | 15.78(3.50-34.73) |
| Yemen | 0.35(0.05-0.85) | 8.93(1.64-21.60) | 0.42(0.03-1.13) | 8.52(1.56-20.56) |
| Bhutan | 0.45(0.05-1.10) | 11.02(1.24-26.25) | 0.88(0.00-2.42) | 10.14(1.27-23.43) |
| Nepal | 2.20(0.36-5.04) | 53.28(9.54-120.73) | 2.92(0.36-6.96) | 50.36(8.61-114.94) |
| India | 2.13(0.51-4.49) | 53.43(13.27-108.47) | 3.18(0.50-7.30) | 50.25(11.87-102.22) |
| Bangladesh | 0.34(0.01-0.93) | 8.91(0.01-23.67) | 0.46(0.00-1.35) | 8.45(0.00-22.62) |
| Pakistan | 0.60(0.10-1.34) | 15.72(2.83-35.82) | 1.20(0.12-3.02) | 14.53(2.73-33.56) |
| Central African Republic | 5.58(0.95-12.37) | 128.36(15.76-294.55) | 4.14(0.22-10.70) | 124.22(15.01-285.38) |
| Democratic Republic of the Congo | 4.66(0.75-10.36) | 101.63(14.09-230.61) | 4.69(0.34-12.23) | 96.94(14.22-223.05) |
| Congo | 10.18(2.18-20.37) | 216.69(41.72-427.38) | 12.60(0.63-30.62) | 204.09(41.17-409.34) |
| Angola | 6.78(1.43-13.74) | 158.21(32.35-319.44) | 8.70(0.71-21.09) | 149.52(29.83-300.26) |
| Gabon | 8.84(1.98-17.51) | 192.18(42.22-385.81) | 13.03(1.02-30.69) | 179.15(40.31-356.39) |
| Equatorial Guinea | 7.04(1.50-14.91) | 148.25(30.79-311.87) | 10.04(0.61-24.34) | 138.21(31.65-290.40) |
| Burundi | 7.24(1.42-14.75) | 172.92(29.39-348.08) | 7.70(0.66-17.60) | 165.22(27.37-331.39) |
| Eritrea | 1.89(0.29-4.27) | 46.84(4.90-110.58) | 1.83(-0.18-5.00) | 45.01(4.83-105.30) |
| Comoros | 0.63(0.12-1.51) | 14.06(2.27-34.03) | 0.90(0.04-2.48) | 13.16(2.04-31.50) |
| Ethiopia | 3.56(0.71-7.34) | 82.40(14.80-173.24) | 4.82(0.33-11.28) | 77.58(13.82-162.44) |
| Djibouti | 0.25(0.01-0.69) | 5.08(-1.42-15.93) | 0.23(-0.44-1.14) | 4.85(-1.08-15.07) |
| Madagascar | 4.33(0.83-9.45) | 105.41(17.45-242.52) | 4.71(0.15-11.49) | 100.71(15.21-234.94) |
| Mauritius | 3.02(0.64-5.68) | 80.82(16.21-151.69) | 7.58(0.82-16.41) | 73.24(16.52-137.27) |
| Rwanda | 7.25(1.47-14.41) | 169.58(34.59-346.23) | 8.58(0.77-19.71) | 161.01(30.97-335.20) |
| Malawi | 4.81(1.05-9.13) | 117.92(26.17-232.22) | 5.03(0.36-11.79) | 112.89(24.89-224.66) |
| Kenya | 5.06(1.11-9.59) | 116.33(25.41-219.76) | 7.33(0.58-16.84) | 109.00(22.65-209.37) |
| Somalia | 0.00(0.00-0.00) | 0.00(0.00-0.00) | 0.00(0.00-0.00) | 0.00(0.00-0.00) |
| Seychelles | 2.83(0.62-5.44) | 80.60(19.42-155.01) | 8.21(1.00-18.84) | 72.39(17.28-136.94) |
| Mozambique | 6.28(1.29-12.84) | 160.52(29.37-338.07) | 5.08(0.24-12.91) | 155.44(28.19-328.35) |
| Uganda | 6.01(1.28-12.07) | 149.00(29.71-304.18) | 9.65(0.83-22.36) | 139.35(25.45-289.28) |
| Zambia | 8.89(2.02-18.11) | 204.88(42.33-421.73) | 9.45(0.82-22.17) | 195.43(39.03-406.58) |
| United Republic of Tanzania | 6.20(1.55-11.99) | 133.67(31.76-262.10) | 10.16(0.60-24.46) | 123.50(28.63-244.22) |
| Botswana | 3.10(0.66-6.23) | 75.45(16.34-148.36) | 6.32(0.51-15.56) | 69.13(15.75-136.81) |
| Namibia | 12.28(2.68-24.77) | 256.34(59.64-502.31) | 13.64(0.95-32.99) | 242.71(56.18-481.00) |
| Lesotho | 8.44(1.70-17.50) | 213.50(40.01-449.88) | 6.03(0.67-14.67) | 207.46(39.42-441.79) |
| Zimbabwe | 6.52(1.46-12.79) | 149.35(29.96-287.67) | 5.77(0.24-13.91) | 143.58(28.45-279.69) |
| South Africa | 5.43(1.26-10.83) | 124.85(30.57-240.92) | 8.99(0.68-21.90) | 115.86(27.40-225.50) |
| Eswatini | 6.37(1.51-12.75) | 147.69(31.40-297.64) | 6.37(0.54-15.17) | 141.32(28.45-288.28) |
| Cabo Verde | 6.79(1.23-13.68) | 156.33(31.51-306.06) | 10.44(1.20-22.78) | 145.89(30.51-285.03) |
| Benin | 4.27(0.82-8.88) | 101.75(20.74-209.75) | 5.84(0.60-13.59) | 95.91(19.94-196.06) |
| Cameroon | 12.15(2.89-23.90) | 278.45(64.42-532.42) | 14.84(1.70-32.28) | 263.62(60.44-505.77) |
| Côte d'Ivoire | 11.72(2.35-23.48) | 263.18(58.60-504.14) | 14.76(1.54-32.36) | 248.42(56.63-475.79) |
| Ghana | 10.73(2.19-21.69) | 254.78(56.32-507.94) | 15.02(1.25-34.87) | 239.75(54.52-479.10) |
| Guinea-Bissau | 9.66(2.24-18.66) | 237.05(55.94-462.56) | 9.15(1.01-20.99) | 227.90(53.02-447.94) |
| Chad | 6.73(1.05-16.02) | 161.07(26.08-375.94) | 7.73(0.42-19.85) | 153.34(26.10-360.77) |
| Gambia | 7.40(1.45-15.32) | 169.84(35.17-343.61) | 8.17(0.75-18.67) | 161.66(34.81-324.10) |
| Burkina Faso | 7.68(1.66-14.58) | 167.09(39.56-319.60) | 8.36(1.01-18.35) | 158.74(38.62-304.70) |
| Guinea | 3.46(0.54-7.57) | 79.51(14.96-176.55) | 4.05(0.35-9.64) | 75.46(14.22-167.84) |
| Mali | 1.86(0.34-3.99) | 39.31(7.87-82.50) | 2.22(0.24-5.33) | 37.09(7.61-77.85) |
| Niger | 0.61(0.06-1.75) | 12.84(0.91-36.49) | 0.58(-0.16-2.24) | 12.26(0.92-34.30) |
| Sao Tome and Principe | 8.08(1.63-15.79) | 185.05(39.84-359.29) | 14.66(1.37-33.11) | 170.39(38.44-326.10) |
| Liberia | 6.72(1.29-12.90) | 160.20(36.45-302.78) | 8.19(0.87-18.98) | 152.01(34.16-289.51) |
| Nigeria | 6.16(1.17-12.62) | 129.29(25.80-257.40) | 9.68(0.90-21.88) | 119.62(24.43-237.21) |
| Mauritania | 0.00(0.00-0.00) | 0.00(0.00-0.00) | 0.00(0.00-0.00) | 0.00(0.00-0.00) |
| Sierra Leone | 5.66(1.15-11.47) | 136.54(28.74-263.20) | 7.57(0.75-16.92) | 128.97(27.08-249.13) |
| Cook Islands | 4.73(0.95-9.59) | 129.85(25.95-258.81) | 17.59(2.54-38.73) | 112.27(18.96-233.59) |
| Togo | 5.40(1.18-11.31) | 131.68(30.09-269.85) | 6.58(0.55-16.33) | 125.10(28.94-258.85) |
| American Samoa | 0.46(0.01-1.49) | 12.60(0.07-42.87) | 1.02(-0.04-3.72) | 11.57(0.07-40.89) |
| Bermuda | 2.04(0.37-4.38) | 44.47(9.06-92.46) | 4.50(0.37-10.52) | 39.97(8.55-82.88) |
| Greenland | 4.58(0.89-9.90) | 101.95(22.08-206.71) | 13.06(0.70-31.93) | 88.89(19.45-180.77) |
| Senegal | 1.09(0.13-2.51) | 23.59(3.02-56.42) | 1.16(-0.08-3.41) | 22.43(3.01-53.66) |
| Nauru | 9.79(1.73-20.82) | 285.97(49.32-608.13) | 14.96(2.01-33.25) | 271.01(39.59-573.52) |
| Northern Mariana Islands | 2.77(0.15-6.84) | 73.88(3.15-183.35) | 6.73(0.28-17.60) | 67.15(2.58-167.60) |
| Guam | 1.49(0.19-3.64) | 51.99(6.70-129.19) | 8.25(0.50-22.42) | 43.74(4.38-106.11) |
| Monaco | 2.96(0.02-8.09) | 53.32(0.02-139.75) | 7.83(-0.30-23.60) | 45.48(0.04-116.35) |
| Niue | 5.11(0.73-11.06) | 142.66(19.48-313.56) | 11.73(1.11-26.72) | 130.93(16.97-295.87) |
| San Marino | 1.98(-0.01-5.01) | 39.81(-0.28-97.10) | 9.23(-0.30-24.17) | 30.58(-0.11-75.06) |
| Palau | 5.41(0.87-12.51) | 153.76(23.32-363.29) | 12.29(1.05-29.37) | 141.47(19.88-330.66) |
| Tuvalu | 3.27(0.51-7.31) | 93.51(13.69-209.11) | 5.36(0.79-12.60) | 88.16(11.36-200.52) |
| Saint Kitts and Nevis | 3.01(0.00-9.29) | 68.44(-0.20-196.40) | 2.41(-0.10-8.23) | 66.02(-0.13-189.62) |
| Tokelau | 3.46(0.55-7.55) | 91.39(12.51-201.41) | 6.21(0.93-14.18) | 85.18(10.51-186.79) |
| Puerto Rico | 0.71(0.17-1.49) | 18.71(4.08-38.12) | 2.26(0.22-5.40) | 16.45(3.71-33.28) |
| United States Virgin Islands | 2.15(0.02-5.46) | 47.15(-0.26-115.80) | 3.62(-0.03-10.29) | 43.54(-0.25-106.12) |
| South Sudan | 0.26(-0.03-0.86) | 5.41(-2.79-19.89) | 0.16(-0.37-0.95) | 5.25(-2.56-19.04) |
| Sudan | 0.00(-0.00-0.00) | -0.01(-0.11-0.03) | -0.00(-0.02-0.00) | -0.01(-0.09-0.03) |
