# Supplementary material for: The global burden of stroke attributable to high alcohol use from 1990 to 2021: An analysis for the global burden of disease study 2021
Source: PLoS One. 2025 Jul 14;20(7):e0328135. doi: 10.1371/journal.pone.0328135 (PMC12258592; doi:10.1371/journal.pone.0328135)
Supplement: S3 Table — (DOCX) [file pone.0328135.s003.docx]

**S3 Table:** ASMR, ASDR, Age-Standardized Rate of YLDs, and Age-Standardized Rate of YLLs of high alcohol use-related Stroke in males and females across 21 GBD regions and SDI regions in 2021. ASMR, age-standardized mortality rate; DALYs, disability-adjusted life years; ASDR, age-standardized rate of DALYs; YLDs, years lived with disability; YLLs, years of life lost.

| **Location Name** | **ASMR**  **per 100,000, N (95% UI)** | | **ASDR**  **per 100,000, N (95% UI)** | | **Age-Standardized Rate of YLDs**  **per 100,000, N (95% UI)** | | **Age-Standardized Rate of YLLs**  **per 100,000, N (95% UI)** | |
| --- | --- | --- | --- | --- | --- | --- | --- | --- |
|  | **Male** | **Female** | **Male** | **Female** | **Male** | **Female** | **Male** | **Female** |
| Andean Latin America | 2.81(0.60-5.47) | 0.58(0.12-1.26) | 67.63(15.08-131.70) | 14.45(2.51-30.55) | 4.41(0.37-10.00) | 1.48(0.03-3.75) | 63.21(14.34-123.83) | 12.97(2.27-28.08) |
| Australasia | 2.95(0.46-6.05) | 1.54(0.22-3.25) | 60.60(10.38-120.31) | 24.98(3.61-52.68) | 16.09(-0.23-35.92) | 5.43(-0.04-13.73) | 44.51(9.34-86.17) | 19.55(3.25-39.81) |
| Caribbean | 5.83(1.42-11.58) | 0.95(0.20-1.95) | 142.59(33.45-282.86) | 26.24(4.70-54.59) | 5.33(0.59-12.14) | 1.66(0.15-4.15) | 137.27(30.89-273.15) | 24.58(4.39-52.25) |
| Central Asia | 9.83(1.69-22.52) | 1.26(0.16-3.13) | 237.76(47.41-500.04) | 30.65(5.11-70.94) | 17.93(1.82-43.49) | 4.21(0.21-11.29) | 219.84(46.02-463.27) | 26.45(4.65-60.30) |
| Central Europe | 14.33(2.03-29.58) | 2.99(0.30-6.88) | 288.72(47.29-568.28) | 56.93(6.92-126.68) | 25.74(-0.18-57.08) | 7.45(-0.02-19.83) | 262.97(44.24-513.41) | 49.48(6.38-109.05) |
| Central Latin America | 2.32(0.51-4.63) | 0.24(0.05-0.60) | 56.10(13.00-109.92) | 6.15(1.23-14.05) | 3.77(0.34-9.26) | 0.83(0.05-2.26) | 52.33(12.76-103.83) | 5.32(1.13-11.88) |
| Central Sub-Saharan Africa | 9.27(1.77-18.73) | 2.89(0.57-6.10) | 201.10(35.40-409.65) | 62.25(11.92-130.63) | 8.45(0.77-19.98) | 4.34(0.15-11.43) | 192.65(34.38-399.07) | 57.91(10.90-124.67) |
| East Asia | 16.69(3.85-32.07) | 1.01(0.20-2.14) | 342.69(83.91-643.57) | 20.75(4.26-42.56) | 26.23(2.55-57.06) | 2.63(0.18-6.58) | 316.46(80.08-591.68) | 18.13(3.86-37.32) |
| Eastern Europe | 13.81(1.40-33.97) | 2.55(0.21-6.74) | 335.09(52.43-744.45) | 62.12(5.65-153.74) | 24.67(0.90-61.84) | 8.55(0.16-23.76) | 310.42(51.45-685.49) | 53.57(5.93-130.34) |
| Eastern Sub-Saharan Africa | 8.53(1.83-16.13) | 1.89(0.42-3.88) | 195.82(41.83-378.06) | 42.67(8.96-86.33) | 9.69(0.82-21.86) | 3.72(0.20-9.41) | 186.13(37.67-363.83) | 38.95(8.59-79.79) |
| High SDI | 3.99(0.74-7.98) | 1.32(0.21-2.81) | 91.44(17.84-180.11) | 27.80(4.36-59.19) | 18.85(0.90-42.28) | 7.63(0.37-18.42) | 72.59(15.84-140.41) | 20.17(3.49-40.87) |
| High-income Asia Pacific | 3.82(0.78-7.35) | 0.90(0.18-1.89) | 94.04(18.12-176.81) | 21.44(3.04-44.35) | 23.41(2.14-50.74) | 7.27(0.57-17.34) | 70.63(14.79-129.23) | 14.17(2.33-28.39) |
| High-income North America | 2.57(0.53-5.52) | 1.39(0.23-3.01) | 64.53(13.65-131.15) | 32.30(5.12-68.45) | 15.52(0.74-37.66) | 9.38(0.23-23.06) | 49.02(11.75-97.46) | 22.92(4.38-46.13) |
| High-middle SDI | 12.09(2.42-24.43) | 1.66(0.22-3.75) | 258.86(59.17-507.42) | 33.15(5.06-73.65) | 22.61(1.79-50.31) | 4.37(0.18-11.20) | 236.25(57.91-458.69) | 28.78(4.77-62.98) |
| Low SDI | 5.59(1.31-10.62) | 1.34(0.29-2.68) | 130.39(30.20-249.39) | 30.43(6.25-60.61) | 6.28(0.78-14.01) | 2.33(0.16-5.61) | 124.12(28.20-238.61) | 28.11(5.88-55.84) |
| Low-middle SDI | 4.77(1.18-9.34) | 0.60(0.14-1.20) | 117.41(27.70-226.51) | 14.80(3.13-29.36) | 6.09(0.90-13.47) | 1.13(0.09-2.66) | 111.32(26.10-215.29) | 13.67(2.89-27.01) |
| Middle SDI | 10.72(2.60-20.41) | 0.77(0.17-1.58) | 233.73(61.33-432.96) | 16.93(3.87-33.89) | 15.49(1.64-33.42) | 1.87(0.14-4.54) | 218.24(56.49-402.75) | 15.06(3.35-30.50) |
| North Africa and Middle East | 0.65(0.10-1.52) | 0.08(0.01-0.20) | 15.38(2.72-34.43) | 1.71(0.25-4.01) | 1.54(0.11-4.22) | 0.20(0.01-0.55) | 13.84(2.51-31.01) | 1.51(0.23-3.61) |
| Oceania | 3.47(0.54-7.70) | 0.32(0.06-0.74) | 91.46(12.79-204.75) | 8.57(1.25-19.78) | 5.19(0.75-12.19) | 0.64(0.06-1.62) | 86.27(11.55-195.40) | 7.92(1.09-18.35) |
| South Asia | 3.60(0.82-7.55) | 0.23(0.05-0.46) | 88.08(21.70-180.70) | 5.89(1.28-11.86) | 5.21(0.82-12.02) | 0.38(0.05-0.90) | 82.88(19.89-170.91) | 5.52(1.17-10.96) |
| Southeast Asia | 10.60(2.67-19.39) | 0.70(0.17-1.37) | 254.45(62.48-471.37) | 17.38(3.71-34.10) | 14.23(2.00-29.40) | 1.81(0.16-4.16) | 240.22(59.83-447.04) | 15.57(3.28-30.15) |
| Southern Latin America | 5.16(1.07-9.96) | 1.66(0.31-3.44) | 115.46(26.73-215.07) | 35.21(6.33-72.10) | 14.93(1.37-32.84) | 6.31(0.52-15.08) | 100.53(24.58-185.95) | 28.90(5.56-56.93) |
| Southern Sub-Saharan Africa | 10.04(2.37-19.07) | 2.97(0.59-6.32) | 229.26(55.65-437.34) | 62.09(14.85-130.41) | 12.70(1.10-29.66) | 5.77(0.03-15.16) | 216.56(51.05-410.53) | 56.32(12.85-118.36) |
| Tropical Latin America | 4.63(0.89-8.96) | 0.83(0.16-1.80) | 105.02(22.98-197.46) | 19.83(4.57-40.82) | 5.55(0.37-13.05) | 1.56(0.10-3.96) | 99.47(22.33-187.48) | 18.28(4.10-37.75) |
| Western Europe | 4.03(0.75-7.92) | 1.57(0.25-3.26) | 80.40(14.73-155.30) | 28.32(4.33-59.47) | 16.81(0.40-37.11) | 6.66(0.09-16.30) | 63.58(12.92-119.88) | 21.66(3.73-43.28) |
