# Supplementary material for: The global burden of stroke attributable to high alcohol use from 1990 to 2021: An analysis for the global burden of disease study 2021
Source: PLoS One. 2025 Jul 14;20(7):e0328135. doi: 10.1371/journal.pone.0328135 (PMC12258592; doi:10.1371/journal.pone.0328135)
Supplement: S4 Table — (DOCX) [file pone.0328135.s004.docx]

**S4 Table:** The number of Death, DALYs, YLDs, and YLLs of high alcohol use-related Stroke in different age groups in 2021. DALYs, disability-adjusted life years; YLDs, years lived with disability; YLLs, years of life lost.

|  | **Death (95% UI)** | | **DALYs (95% UI)** | | **YLDs (95% UI)** | | **YLLs (95% UI)** | |
| --- | --- | --- | --- | --- | --- | --- | --- | --- |
| **Age Groups** | **Male** | **Female** | **Male** | **Female** | **Male** | **Female** | **Male** | **Female** |
| 15-19 years | -34.53(58.17--74.19) | -5.91(20.51--27.47) | -3085.06(4870.23--6480.60) | -761.88(1948.77--2818.12) | -580.96(586.86--1295.20) | -333.47(480.35--936.51) | -2504.11(4216.45--5380.15) | -428.41(1487.82--1993.01) |
| 20-24 years | 72.48(492.49--22.07) | 12.14(78.78--29.00) | 5648.17(38987.16--1831.69) | 915.94(8411.03--2927.40) | 750.48(5804.10--418.13) | 94.63(3061.56--1135.31) | 4897.68(33299.93--1494.03) | 821.31(5329.25--1962.16) |
| 25-29 years | 276.17(925.94--18.67) | 29.46(115.95--24.70) | 20213.24(67667.60--1617.59) | 2667.85(12303.51--2627.54) | 2922.30(12547.12--490.95) | 821.28(5436.21--1263.85) | 17290.94(58005.33--1170.00) | 1846.57(7266.25--1548.36) |
| 30-34 years | 1194.75(2742.72--22.30) | 95.17(246.73--22.20) | 78230.84(175095.79--1460.30) | 7858.73(21631.20--2616.27) | 9343.93(23952.90--316.66) | 2366.31(8234.13--1174.29) | 68886.91(158157.84--1290.88) | 5492.42(14235.42--1280.96) |
| 35-39 years | 2545.22(5224.45-89.63) | 211.72(470.88--14.65) | 150475.19(304510.24-6405.89) | 15133.03(34038.67--1766.33) | 16097.36(35373.50-893.38) | 3956.70(10745.55--906.00) | 134377.83(275829.78-4733.07) | 11176.32(24857.15--772.90) |
| 40-44 years | 5194.84(10168.10-610.57) | 471.00(970.99-18.70) | 272847.50(524169.60-38874.60) | 28874.97(61863.22-1717.86) | 24322.94(48869.25-3121.05) | 6336.01(15161.72--20.33) | 248524.56(486474.80-29209.74) | 22538.95(46466.62-893.44) |
| 45-49 years | 9168.83(17380.65-1516.49) | 917.03(1836.97-145.44) | 433085.19(822841.55-83796.03) | 48861.35(96327.92-8248.43) | 39510.13(78969.17-5392.91) | 9461.75(22241.18-651.80) | 393575.05(746186.13-65096.17) | 39399.60(78923.03-6248.41) |
| 50-54 years | 15970.47(30271.90-3287.45) | 1566.78(3068.77-278.33) | 667922.20(1258959.71-158964.77) | 72861.79(140296.65-16104.70) | 58191.89(120682.61-8350.22) | 13040.74(30539.54-1049.59) | 609730.31(1155788.72-125496.37) | 59821.04(117157.03-10626.67) |
| 55-59 years | 22746.50(42754.20-5314.31) | 2305.89(4421.83-532.81) | 837816.71(1562688.54-207347.78) | 94282.42(178895.16-23418.45) | 75165.38(160582.27-10062.48) | 17037.61(38286.85-1497.29) | 762651.33(1433465.11-178148.24) | 77244.81(148126.87-17839.87) |
| 60-64 years | 29995.57(55750.26-8081.27) | 3490.54(6961.54-822.74) | 945928.46(1752589.73-247487.04) | 120903.91(242925.13-29263.40) | 80285.50(178172.32-8544.71) | 20188.18(49351.22-870.13) | 865642.95(1608883.72-233186.79) | 100715.73(200882.97-23734.94) |
| 65-69 years | 40489.92(76565.53-10479.76) | 4756.34(9554.27-1072.92) | 1076522.07(2034547.80-276016.66) | 137141.99(279446.96-29135.50) | 92196.31(209736.51-2438.13) | 21621.90(53475.35-276.37) | 984325.75(1861139.48-254761.98) | 115520.09(232065.19-26057.00) |
| 70-74 years | 47497.37(92168.98-10586.58) | 6516.65(13197.07-1140.54) | 1041026.62(2045110.49-225019.76) | 152942.36(316056.79-24580.76) | 90691.71(212844.19--649.54) | 22760.27(57179.58-53.45) | 950334.90(1844394.17-211705.06) | 130182.08(263677.00-22774.53) |
| 75-79 years | 43647.29(86956.04-8698.55) | 7241.32(15803.19-1305.68) | 769223.11(1527813.72-146943.82) | 133625.39(290976.66-20707.35) | 71792.44(164422.76--1285.97) | 18484.34(45500.63--11.68) | 697430.67(1389398.62-138988.01) | 115141.06(251264.42-20772.62) |
| 80-84 years | 36656.63(76064.27-5826.52) | 8606.05(19118.53-958.24) | 505378.19(1068789.37-79006.44) | 122564.39(272768.56-11226.85) | 48219.41(113574.87--1017.42) | 15676.27(37146.86--52.11) | 457158.77(948861.50-72654.97) | 106888.12(237489.60-11904.42) |
| 85-89 years | 31023.38(65388.11-4227.11) | 9391.66(20939.48-822.26) | 331174.43(704950.43-41827.17) | 104584.30(234490.82-7707.92) | 23909.93(56886.31--293.56) | 11985.75(28788.27-212.63) | 307264.50(647824.16-41851.52) | 92598.56(206550.18-8108.25) |
| 90-94 years | 13450.10(29370.39-1566.45) | 7620.71(17296.62-428.57) | 124096.14(270630.83-14023.25) | 72164.77(166906.11-3839.93) | 7993.59(19349.69-87.78) | 6568.52(15986.09-162.44) | 116102.54(253515.81-13537.37) | 65596.25(148893.23-3688.88) |
| 95+ years | 3344.26(7583.02-294.52) | 4253.83(10173.11-186.50) | 29265.83(66782.39-2400.60) | 36882.19(88769.14-1602.21) | 2184.21(5364.66-44.44) | 2668.93(6737.16-69.48) | 27081.63(61407.06-2412.74) | 34213.26(81818.99-1499.68) |
