# Supplementary material for: The global burden of stroke attributable to high alcohol use from 1990 to 2021: An analysis for the global burden of disease study 2021
Source: PLoS One. 2025 Jul 14;20(7):e0328135. doi: 10.1371/journal.pone.0328135 (PMC12258592; doi:10.1371/journal.pone.0328135)
Supplement: S5 Table — (DOCX) [file pone.0328135.s005.docx]

**S5 Table:** The rate of Death, DALYs, YLDs, and YLLs of high alcohol use-related Stroke in different age groups in 2021. DALYs, disability-adjusted life years; YLDs, years lived with disability; YLLs, years of life lost.

|  | **Death Rate** **per 100,000, N (95% UI)** | | **DALYs Rate** **per 100,000, N (95% UI)** | | **YLDS Rate** **per 100,000, N (95% UI)** | | **YLLS Rate** **per 100,000, N (95% UI)** | |
| --- | --- | --- | --- | --- | --- | --- | --- | --- |
| **Age Groups** | **Male** | **Female** | **Male** | **Female** | **Male** | **Female** | **Male** | **Female** |
| 15-19 years | -0.01(0.02--0.02) | 0.00(0.01--0.01) | -0.96(1.52--2.02) | -0.25(0.64--0.93) | -0.18(0.18--0.40) | -0.11(0.16--0.31) | -0.78(1.32--1.68) | -0.14(0.49--0.66) |
| 20-24 years | 0.02(0.16--0.01) | 0.00(0.03--0.01) | 1.86(12.85--0.60) | 0.31(2.86--1.00) | 0.25(1.91--0.14) | 0.03(1.04--0.39) | 1.61(10.98--0.49) | 0.28(1.81--0.67) |
| 25-29 years | 0.09(0.31--0.01) | 0.01(0.04--0.01) | 6.80(22.76--0.54) | 0.92(4.23--0.90) | 0.98(4.22--0.17) | 0.28(1.87--0.43) | 5.81(19.51--0.39) | 0.63(2.50--0.53) |
| 30-34 years | 0.39(0.90--0.01) | 0.03(0.08--0.01) | 25.60(57.31--0.48) | 2.63(7.24--0.88) | 3.06(7.84--0.10) | 0.79(2.75--0.39) | 22.55(51.76--0.42) | 1.84(4.76--0.43) |
| 35-39 years | 0.90(1.85-0.03) | 0.08(0.17--0.01) | 53.16(107.58-2.26) | 5.45(12.25--0.64) | 5.69(12.50-0.32) | 1.42(3.87--0.33) | 47.47(97.44-1.67) | 4.02(8.95--0.28) |
| 40-44 years | 2.06(4.03-0.24) | 0.19(0.39-0.01) | 108.20(207.87-15.42) | 11.64(24.94-0.69) | 9.65(19.38-1.24) | 2.55(6.11--0.01) | 98.56(192.92-11.58) | 9.08(18.73-0.36) |
| 45-49 years | 3.85(7.31-0.64) | 0.39(0.78-0.06) | 182.07(345.93-35.23) | 20.74(40.88-3.50) | 16.61(33.20-2.27) | 4.02(9.44-0.28) | 165.46(313.71-27.37) | 16.72(33.49-2.65) |
| 50-54 years | 7.19(13.64-1.48) | 0.70(1.38-0.12) | 300.89(567.15-71.61) | 32.68(62.93-7.22) | 26.21(54.37-3.76) | 5.85(13.70-0.47) | 274.68(520.67-56.53) | 26.83(52.55-4.77) |
| 55-59 years | 11.68(21.96-2.73) | 1.15(2.20-0.27) | 430.26(802.51-106.48) | 46.91(89.00-11.65) | 38.60(82.47-5.17) | 8.48(19.05-0.74) | 391.66(736.15-91.49) | 38.43(73.69-8.88) |
| 60-64 years | 19.29(35.84-5.20) | 2.12(4.23-0.50) | 608.17(1126.80-159.12) | 73.49(147.67-17.79) | 51.62(114.55-5.49) | 12.27(30.00-0.53) | 556.55(1034.41-149.92) | 61.22(122.11-14.43) |
| 65-69 years | 30.71(58.08-7.95) | 3.30(6.63-0.75) | 816.58(1543.27-209.37) | 95.23(194.05-20.23) | 69.93(159.09-1.85) | 15.01(37.13-0.19) | 746.64(1411.74-193.25) | 80.22(161.15-18.09) |
| 70-74 years | 49.28(95.62-10.98) | 5.95(12.06-1.04) | 1080.00(2121.67-233.44) | 139.74(288.77-22.46) | 94.09(220.81--0.67) | 20.80(52.24-0.05) | 985.91(1913.44-219.63) | 118.94(240.92-20.81) |
| 75-79 years | 73.00(145.44-14.55) | 10.04(21.92-1.81) | 1286.61(2555.44-245.78) | 185.34(403.59-28.72) | 120.08(275.02--2.15) | 25.64(63.11--0.02) | 1166.53(2323.93-232.47) | 159.70(348.51-28.81) |
| 80-84 years | 100.01(207.53-15.90) | 16.90(37.54-1.88) | 1378.86(2916.06-215.56) | 240.65(535.56-22.04) | 131.56(309.87--2.78) | 30.78(72.94--0.10) | 1247.30(2588.85-198.23) | 209.87(466.29-23.37) |
| 85-89 years | 179.82(379.00-24.50) | 32.99(73.55-2.89) | 1919.56(4086.04-242.44) | 367.36(823.67-27.07) | 138.59(329.72--1.70) | 42.10(101.12-0.75) | 1780.97(3754.92-242.58) | 325.26(725.52-28.48) |
| 90-94 years | 230.76(503.91-26.88) | 63.19(143.41-3.55) | 2129.13(4643.23-240.60) | 598.34(1383.86-31.84) | 137.15(331.98-1.51) | 54.46(132.55-1.35) | 1991.98(4349.59-232.26) | 543.88(1234.51-30.59) |
| 95+ years | 221.18(501.51-19.48) | 108.01(258.31-4.74) | 1935.52(4416.70-158.77) | 936.51(2254.02-40.68) | 144.45(354.80-2.94) | 67.77(171.07-1.76) | 1791.06(4061.20-159.57) | 868.74(2077.54-38.08) |
