# Supplementary material for: The global burden of stroke attributable to high alcohol use from 1990 to 2021: An analysis for the global burden of disease study 2021
Source: PLoS One. 2025 Jul 14;20(7):e0328135. doi: 10.1371/journal.pone.0328135 (PMC12258592; doi:10.1371/journal.pone.0328135)
Supplement: S6 Table — (DOCX) [file pone.0328135.s006.docx]

**S6 Table:** ASMR for two types of high alcohol use-related Stroke in both sexes combined globally, 1990-2021. ASMR, age-standardized mortality rate.

| **Year** | **Ischemic stroke** | **Intracerebral hemorrhage** |
| --- | --- | --- |
| 1990 | 4.15(-0.43-10.20) | 3.05(0.05-6.51) |
| 1991 | 4.06(-0.42-10.04) | 3.02(0.05-6.29) |
| 1992 | 4.00(-0.42-10.03) | 3.02(0.04-6.24) |
| 1993 | 4.02(-0.43-9.91) | 3.04(0.05-6.43) |
| 1994 | 3.99(-0.43-9.97) | 3.03(0.05-6.31) |
| 1995 | 3.94(-0.43-9.78) | 3.02(0.05-6.23) |
| 1996 | 3.81(-0.43-9.44) | 2.96(0.04-6.16) |
| 1997 | 3.68(-0.42-9.10) | 2.89(0.04-5.88) |
| 1998 | 3.57(-0.40-8.88) | 2.83(0.05-5.85) |
| 1999 | 3.50(-0.41-8.63) | 2.79(0.05-5.81) |
| 2000 | 3.42(-0.41-8.43) | 2.79(0.05-5.65) |
| 2001 | 3.37(-0.41-8.30) | 2.78(0.06-5.67) |
| 2002 | 3.35(-0.41-8.29) | 2.77(0.06-5.78) |
| 2003 | 3.31(-0.41-8.16) | 2.78(0.07-5.74) |
| 2004 | 3.19(-0.40-7.82) | 2.75(0.07-5.61) |
| 2005 | 3.10(-0.39-7.64) | 2.69(0.07-5.56) |
| 2006 | 2.91(-0.37-7.22) | 2.57(0.07-5.33) |
| 2007 | 2.82(-0.37-6.89) | 2.52(0.07-5.31) |
| 2008 | 2.79(-0.36-6.82) | 2.53(0.07-5.23) |
| 2009 | 2.73(-0.36-6.61) | 2.52(0.08-5.26) |
| 2010 | 2.69(-0.35-6.59) | 2.51(0.08-5.22) |
| 2011 | 2.61(-0.35-6.43) | 2.45(0.08-5.12) |
| 2012 | 2.55(-0.33-6.28) | 2.40(0.07-5.07) |
| 2013 | 2.49(-0.32-6.09) | 2.34(0.08-4.97) |
| 2014 | 2.43(-0.32-5.95) | 2.28(0.07-4.69) |
| 2015 | 2.39(-0.31-5.98) | 2.25(0.07-4.70) |
| 2016 | 2.36(-0.32-5.85) | 2.22(0.07-4.62) |
| 2017 | 2.31(-0.32-5.67) | 2.17(0.06-4.49) |
| 2018 | 2.28(-0.30-5.58) | 2.14(0.06-4.47) |
| 2019 | 2.25(-0.30-5.60) | 2.12(0.06-4.51) |
| 2020 | 2.23(-0.31-5.42) | 2.10(0.05-4.35) |
| 2021 | 2.21(-0.29-5.53) | 2.09(0.06-4.28) |
