# Supplementary material for: The global burden of stroke attributable to high alcohol use from 1990 to 2021: An analysis for the global burden of disease study 2021
Source: PLoS One. 2025 Jul 14;20(7):e0328135. doi: 10.1371/journal.pone.0328135 (PMC12258592; doi:10.1371/journal.pone.0328135)
Supplement: S7 Table — (DOCX) [file pone.0328135.s007.docx]

**S7 Table:** ASDR for two types of high alcohol use-related Stroke in both sexes combined globally, 1990-2021. DALYs, disability-adjusted life years; ASDR, age-standardized rate of DALYs.

| **Year** | **Ischemic stroke** | **Intracerebral hemorrhage** |
| --- | --- | --- |
| 1990 | 76.45(-9.55-186.84) | 78.37(1.25-164.48) |
| 1991 | 75.32(-9.29-184.52) | 77.72(1.25-160.46) |
| 1992 | 74.90(-9.65-184.65) | 77.66(1.17-161.05) |
| 1993 | 75.86(-9.82-185.31) | 78.22(1.27-161.46) |
| 1994 | 76.05(-9.98-186.65) | 78.11(1.25-159.13) |
| 1995 | 75.04(-9.99-183.59) | 77.61(1.32-159.50) |
| 1996 | 72.69(-9.89-177.61) | 75.96(1.19-154.50) |
| 1997 | 70.21(-9.60-171.16) | 73.82(1.21-148.33) |
| 1998 | 68.31(-9.28-167.72) | 72.06(1.24-146.29) |
| 1999 | 67.17(-9.30-162.43) | 71.11(1.34-145.26) |
| 2000 | 66.03(-9.26-159.63) | 70.96(1.35-142.83) |
| 2001 | 65.29(-9.29-158.78) | 70.50(1.47-143.58) |
| 2002 | 64.89(-9.30-158.49) | 70.36(1.53-141.53) |
| 2003 | 64.33(-9.28-155.14) | 70.34(1.68-144.56) |
| 2004 | 62.40(-9.03-150.41) | 69.58(1.64-141.05) |
| 2005 | 61.07(-9.12-147.94) | 68.30(1.81-140.19) |
| 2006 | 57.72(-8.71-140.85) | 65.51(1.82-135.55) |
| 2007 | 56.07(-8.61-136.15) | 64.45(1.87-132.94) |
| 2008 | 55.63(-8.42-134.90) | 64.56(1.92-132.65) |
| 2009 | 54.55(-8.53-132.50) | 64.04(1.99-132.11) |
| 2010 | 53.88(-8.33-130.76) | 63.42(2.02-131.42) |
| 2011 | 52.48(-8.26-128.99) | 61.95(1.96-126.66) |
| 2012 | 51.46(-8.00-123.72) | 60.84(1.83-125.09) |
| 2013 | 50.31(-7.69-122.56) | 59.25(1.86-123.90) |
| 2014 | 49.32(-7.69-119.94) | 57.88(1.76-116.58) |
| 2015 | 48.59(-7.51-119.89) | 56.95(1.71-118.13) |
| 2016 | 48.09(-7.83-115.93) | 56.30(1.68-116.71) |
| 2017 | 47.15(-7.56-115.92) | 54.92(1.71-112.43) |
| 2018 | 46.62(-7.32-112.97) | 54.12(1.64-112.38) |
| 2019 | 46.13(-7.02-114.29) | 53.53(1.63-113.26) |
| 2020 | 45.76(-7.48-111.84) | 52.94(1.49-108.86) |
| 2021 | 45.44(-6.99-112.92) | 52.45(1.54-108.00) |
