# Supplementary material for: The global burden of stroke attributable to high alcohol use from 1990 to 2021: An analysis for the global burden of disease study 2021
Source: PLoS One. 2025 Jul 14;20(7):e0328135. doi: 10.1371/journal.pone.0328135 (PMC12258592; doi:10.1371/journal.pone.0328135)
Supplement: S8 Table — (DOCX) [file pone.0328135.s008.docx]

**S8 Table:** Age-Standardized Rate of YLDs for two types of high alcohol use-related Stroke in both sexes combined globally, 1990-2021. YLDs, years lived with disability.

| **Year** | **Ischemic stroke** | **Intracerebral hemorrhage** |
| --- | --- | --- |
| 1990 | 8.03(-1.25-20.22) | 2.17(0.04-4.69) |
| 1991 | 8.03(-1.26-20.19) | 2.17(0.04-4.65) |
| 1992 | 8.03(-1.28-20.23) | 2.17(0.04-4.60) |
| 1993 | 8.02(-1.30-20.21) | 2.16(0.04-4.57) |
| 1994 | 8.01(-1.30-20.16) | 2.15(0.04-4.55) |
| 1995 | 7.99(-1.30-20.08) | 2.14(0.04-4.53) |
| 1996 | 7.95(-1.30-19.96) | 2.13(0.04-4.49) |
| 1997 | 7.90(-1.31-19.78) | 2.10(0.04-4.45) |
| 1998 | 7.83(-1.31-19.59) | 2.08(0.04-4.39) |
| 1999 | 7.77(-1.31-19.35) | 2.05(0.04-4.33) |
| 2000 | 7.73(-1.31-19.23) | 2.03(0.03-4.30) |
| 2001 | 7.69(-1.31-19.15) | 2.02(0.03-4.27) |
| 2002 | 7.67(-1.31-19.13) | 2.01(0.03-4.24) |
| 2003 | 7.64(-1.31-19.04) | 2.00(0.03-4.21) |
| 2004 | 7.61(-1.33-18.96) | 1.99(0.04-4.18) |
| 2005 | 7.59(-1.33-18.92) | 1.97(0.04-4.18) |
| 2006 | 7.58(-1.34-18.87) | 1.97(0.04-4.17) |
| 2007 | 7.60(-1.36-18.88) | 1.98(0.04-4.17) |
| 2008 | 7.63(-1.38-18.90) | 1.99(0.05-4.21) |
| 2009 | 7.67(-1.40-18.98) | 2.00(0.05-4.21) |
| 2010 | 7.69(-1.42-19.06) | 2.00(0.06-4.24) |
| 2011 | 7.70(-1.41-19.06) | 1.98(0.06-4.18) |
| 2012 | 7.70(-1.40-19.03) | 1.96(0.05-4.14) |
| 2013 | 7.71(-1.39-19.07) | 1.94(0.05-4.07) |
| 2014 | 7.71(-1.38-19.03) | 1.91(0.05-4.01) |
| 2015 | 7.71(-1.37-19.03) | 1.89(0.05-3.96) |
| 2016 | 7.70(-1.39-18.98) | 1.87(0.05-3.88) |
| 2017 | 7.68(-1.39-18.94) | 1.84(0.05-3.82) |
| 2018 | 7.66(-1.39-18.89) | 1.82(0.05-3.79) |
| 2019 | 7.65(-1.40-18.78) | 1.81(0.05-3.76) |
| 2020 | 7.64(-1.40-18.85) | 1.80(0.05-3.74) |
| 2021 | 7.63(-1.40-18.72) | 1.82(0.05-3.81) |
