# Supplementary material for: The global burden of stroke attributable to high alcohol use from 1990 to 2021: An analysis for the global burden of disease study 2021
Source: PLoS One. 2025 Jul 14;20(7):e0328135. doi: 10.1371/journal.pone.0328135 (PMC12258592; doi:10.1371/journal.pone.0328135)
Supplement: S9 Table — (DOCX) [file pone.0328135.s009.docx]

**S9 Table:** Age-Standardized Rate of YLLs for two types of high alcohol use-related Stroke in both sexes combined globally, 1990-2021. YLLs, years of life lost.

| **Year** | **Ischemic stroke** | **Intracerebral hemorrhage** |
| --- | --- | --- |
| 1990 | 68.42(-8.20-167.83) | 76.20(1.21-160.38) |
| 1991 | 67.29(-7.93-165.39) | 75.55(1.21-156.11) |
| 1992 | 66.87(-8.27-165.94) | 75.50(1.15-155.81) |
| 1993 | 67.84(-8.41-165.88) | 76.06(1.24-157.28) |
| 1994 | 68.04(-8.55-167.18) | 75.95(1.22-155.07) |
| 1995 | 67.05(-8.56-164.16) | 75.46(1.30-154.94) |
| 1996 | 64.74(-8.54-159.55) | 73.83(1.17-150.57) |
| 1997 | 62.31(-8.17-152.89) | 71.72(1.17-144.14) |
| 1998 | 60.48(-7.85-149.19) | 69.98(1.20-142.19) |
| 1999 | 59.40(-7.87-144.49) | 69.06(1.32-141.06) |
| 2000 | 58.30(-7.91-142.57) | 68.93(1.32-138.64) |
| 2001 | 57.59(-7.85-139.94) | 68.48(1.45-139.26) |
| 2002 | 57.22(-7.85-140.13) | 68.35(1.50-137.73) |
| 2003 | 56.69(-7.82-138.61) | 68.35(1.65-140.43) |
| 2004 | 54.79(-7.67-134.04) | 67.59(1.61-137.00) |
| 2005 | 53.48(-7.62-130.66) | 66.33(1.77-136.13) |
| 2006 | 50.14(-7.20-123.80) | 63.54(1.78-131.40) |
| 2007 | 48.47(-7.09-117.00) | 62.47(1.83-129.06) |
| 2008 | 48.00(-6.91-116.21) | 62.57(1.87-128.35) |
| 2009 | 46.88(-6.96-113.33) | 62.04(1.94-128.05) |
| 2010 | 46.19(-6.74-111.77) | 61.42(1.96-127.28) |
| 2011 | 44.78(-6.69-110.02) | 59.96(1.90-122.94) |
| 2012 | 43.76(-6.44-106.56) | 58.87(1.77-121.21) |
| 2013 | 42.60(-6.13-104.25) | 57.31(1.81-120.74) |
| 2014 | 41.61(-6.25-101.91) | 55.97(1.71-112.83) |
| 2015 | 40.88(-6.07-102.30) | 55.06(1.66-114.08) |
| 2016 | 40.40(-6.29-98.05) | 54.43(1.62-113.15) |
| 2017 | 39.47(-6.16-97.37) | 53.08(1.66-108.81) |
| 2018 | 38.95(-5.85-94.03) | 52.30(1.59-108.51) |
| 2019 | 38.48(-5.88-94.35) | 51.71(1.58-108.97) |
| 2020 | 38.12(-6.09-92.75) | 51.13(1.44-105.04) |
| 2021 | 37.80(-5.61-94.02) | 50.64(1.49-103.79) |
