# Supplementary figures and images for: The global burden of stroke attributable to high alcohol use from 1990 to 2021: An analysis for the global burden of disease study 2021
Source: PLoS One. 2025 Jul 14;20(7):e0328135. doi: 10.1371/journal.pone.0328135 (PMC12258592; doi:10.1371/journal.pone.0328135)

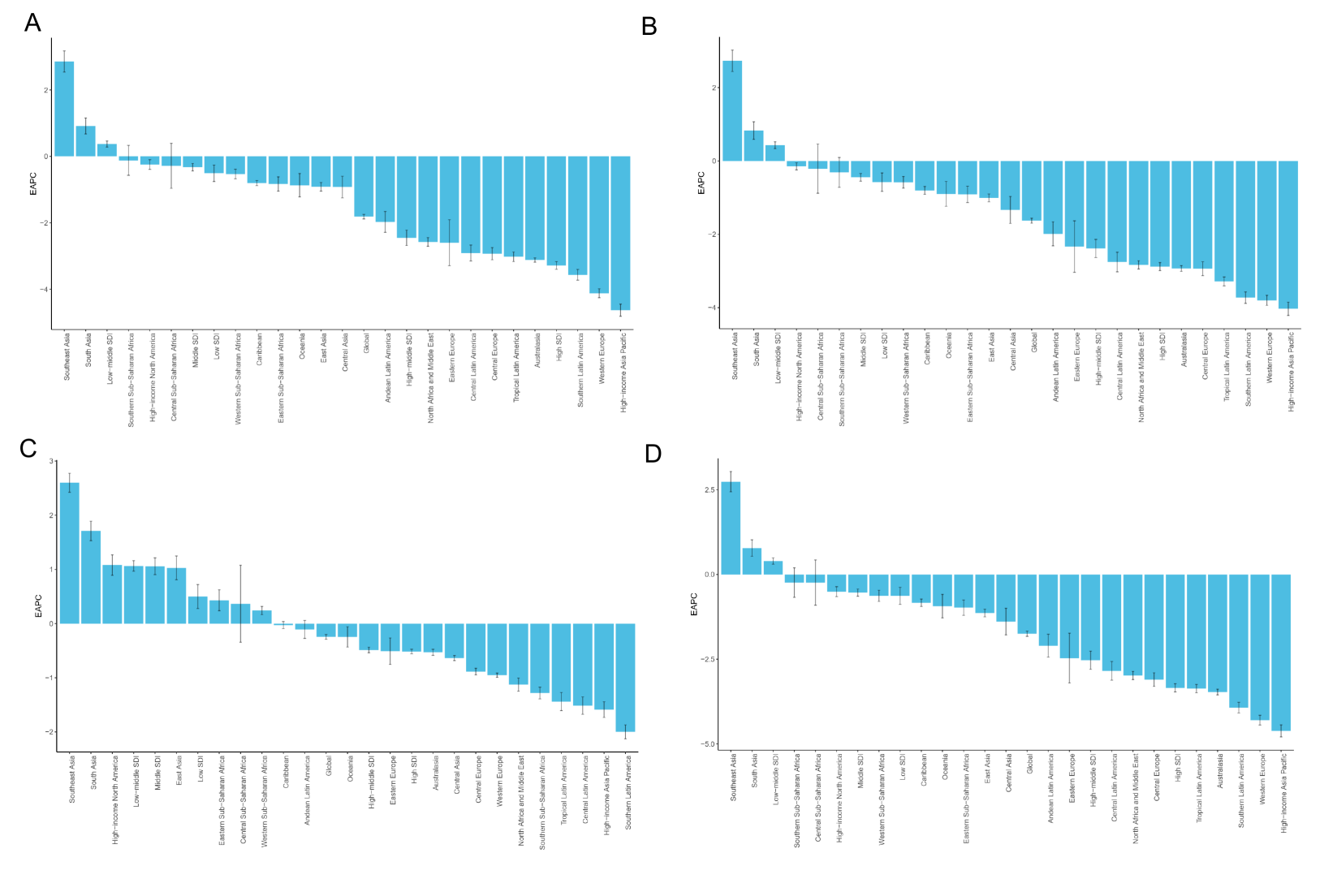

Supplement: S1 Fig — (TIFF) [file pone.0328135.s010.tiff]

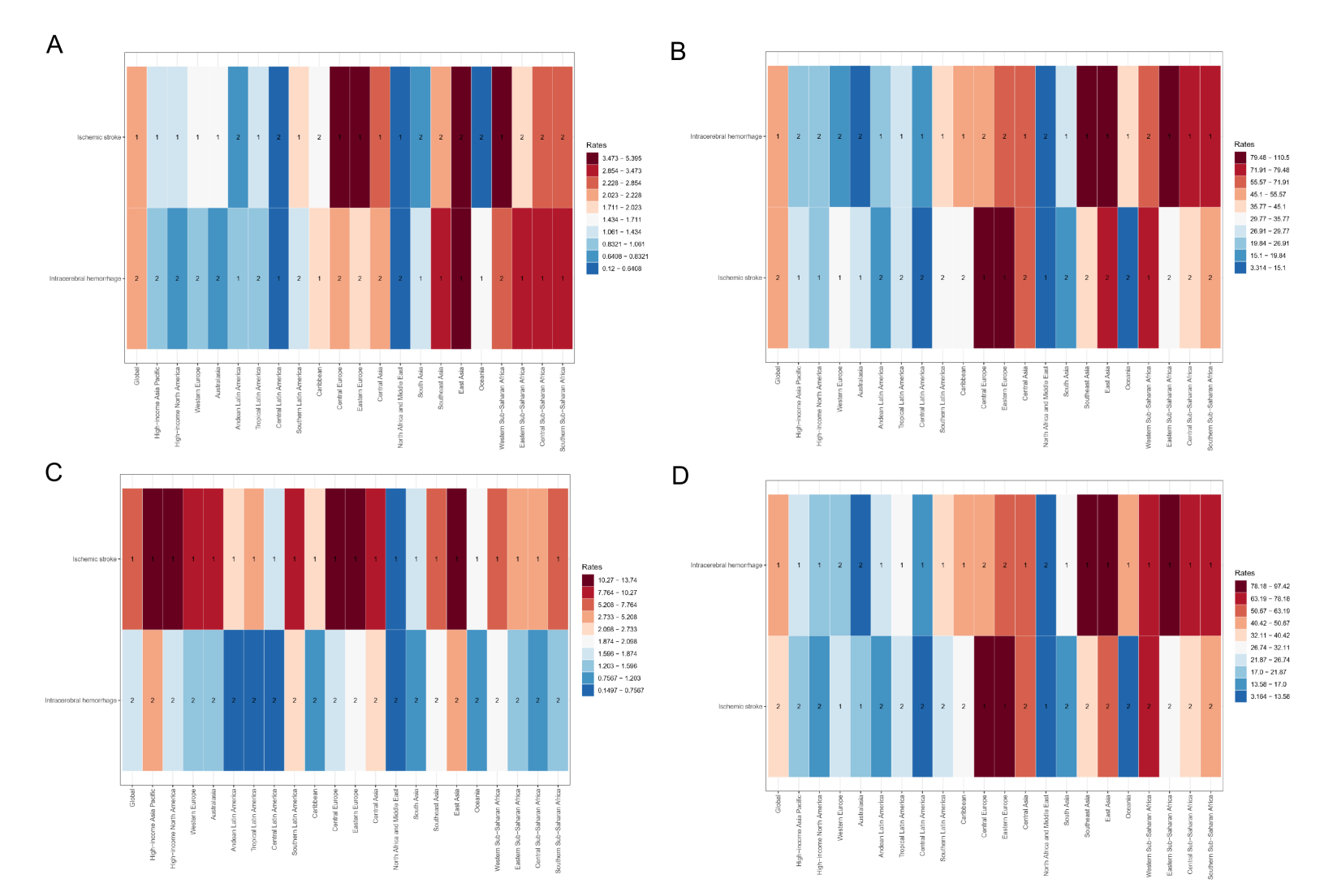

Supplement: S2 Fig — (TIFF) [file pone.0328135.s011.tiff]
